# USP7 inhibitors suppress tumour neoangiogenesis and promote synergy with immune checkpoint inhibitors by downregulating fibroblast VEGF

**DOI:** 10.1002/ctm2.1648

**Published:** 2024-04-11

**Authors:** Anamarija Jurisic, Pei‐Ju Sung, Mark Wappett, Julien Daubriac, Ian T. Lobb, Wei‐Wei Kung, Nyree Crawford, Natalie Page, Eamon Cassidy, Stephanie Feutren‐Burton, J. S. Shane Rountree, Matthew D. Helm, Colin R. O'Dowd, Richard D. Kennedy, Gerald Gavory, Aaron N. Cranston, Daniel B. Longley, Xavier Jacq, Timothy Harrison

**Affiliations:** ^1^ Almac Discovery Ltd., Health Science Building Belfast UK; ^2^ Patrick G Johnston Centre for Cancer Research Queen's University Belfast Belfast UK

**Keywords:** ADC‐159, CAFs, deubiquitylating enzymes, DUBs, HAUSP, HIF‐1α, immune checkpoint inhibitors, immune therapy, IO, neoangiogenesis, TME, tumour microenvironment, USP7, VEGF

## Abstract

**Background:**

Understanding how to modulate the microenvironment of tumors that are resistant to immune checkpoint inhibitors represents a major challenge in oncology.Here we investigate the ability of USP7 inhibitors to reprogram the tumor microenvironment (TME) by inhibiting secretion of vascular endothelial growth factor (VEGF) from fibroblasts.

**Methods:**

To understand the role played by USP7 in the TME, we systematically evaluated the effects of potent, selective USP7 inhibitors on co‐cultures comprising components of the TME, using human primary cells. We also evaluated the effects of USP7 inhibition on tumor growth inhibition in syngeneic models when dosed in combination with immune checkpoint inhibitors (ICIs).

**Results:**

Abrogation of VEGF secretion from fibroblasts in response to USP7 inhibition resulted in inhibition of tumor neoangiogenesis and increased tumor recruitment of CD8‐positive T‐lymphocytes, leading to significantly improved sensitivity to immune checkpoint inhibitors. In syngeneic models, treatment with USP7 inhibitors led to striking tumor responses resulting in significantly improved survival.

**Conclusions:**

USP7‐mediated reprograming of the TME is not linked to its previously characterized role in modulating MDM2 but does require p53 and UHRF1 in addition to the well‐characterized VEGF transcription factor, HIF‐1α. This represents a function of USP7 that is unique to fibroblasts, and which is not observed in cancer cells or other components of the TME. Given the potential for USP7 inhibitors to transform “immune desert” tumors into “immune responsive” tumors, this paves the way for a novel therapeutic strategy combining USP7 inhibitors with immune checkpoint inhibitors (ICIs).

## Graphical abstract

The oral USP7 inhibitor, ADC‐159, reduces sVEGF from CAFs and impacts tumor vasculature. USP7 inhibition affects HIF‐1α transcriptional modulation, tumor hypoxia and remodeling of the tumor microenvironment creating a permissive immune micro‐climate for infiltrating lymphocytes turning immunologically ‘cold’ tumors, ‘hot’. In preclinical models, combination treatment of ADC‐159 with immunotherapy agents delivers improved anti‐tumor efficacy and survival.

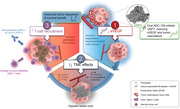

## 1 INTRODUCTION

To enable metastatic disease progression, a number of hallmarks need to be acquired by both cancer cells and components of the tumour microenvironment (TME).^1^ The orchestration of TME changes that occur during tumourigenesis affects the entire stroma including endothelial cells, immune cells, fibroblasts and the extra‐cellular matrix.^2^ Changes in the TME not only facilitate the transition from benign to aggressive tumours, but also contribute to tumour heterogeneity, variation in patient responses and can lead to cancer therapy resistance.^3^ Recent advances in cancer immunotherapy have presented opportunities to treat cancers that were not responsive to previous standard‐of‐care therapies.^4^, ^5^ However, clinical resistance to immunotherapy can be driven by immunosuppressive mechanisms, such as dysfunctional T‐cells, an absence of tumour T‐cell infiltration, or a loss of tumour recognition by T‐cells.^6^

An important factor in the remodelling of the TME is the transcription factor HIF‐1α.^7^ HIF‐1α activates the transcription of genes involved in angiogenesis, metabolism, proliferation/survival and invasion/metastasis.^7^, ^8^, ^9^, ^10^, ^11^, ^12^ HIF‐1α upregulates VEGF transcription in response to hypoxia in solid tumours.^13^, ^14^ Levels of circulating VEGF correlate with decreased disease‐free survival and overall survival in various solid cancers.^15^, ^16^ Our understanding of HIF‐1α stability and p53 modulation is largely driven by studies in endothelial cells or cancer cells, and there is limited information available regarding its regulation in other components of the TME.^17^

In cancer cells, ubiquitin specific protease 7 (USP7, HAUSP) is best known for removing ubiquitin molecules from the oncoprotein MDM2 in the p53 tumour suppressor pathway.^18^, ^19^ To date, however, the role of USP7 in TME biology remains largely unknown. Recently, breakthroughs have been made in the discovery of novel highly potent and selective USP7 inhibitors, providing powerful pharmacological tools to further explore USP7 biology.^20^, ^21^, ^22^

To understand the role played by USP7 in the TME, we systematically evaluated the effects of potent, selective USP7 inhibitors^23^, ^24^ on co‐cultures comprising components of the TME, using human primary cells. We report a critical role for USP7 in regulation of HIF‐1α mediated VEGF production by cancer‐associated fibroblasts (CAFs) leading to reprograming of the TME. USP7 inhibition in fibroblasts leads to a significant decrease in tumour neoangiogenesis, modulation of the tumour immune microenvironment, tumour growth inhibition and striking tumour responses in syngeneic models when dosed in combination with immune checkpoint inhibitors (ICIs), resulting in significantly improved survival.

## 2 RESULTS

**FIGURE 1:**
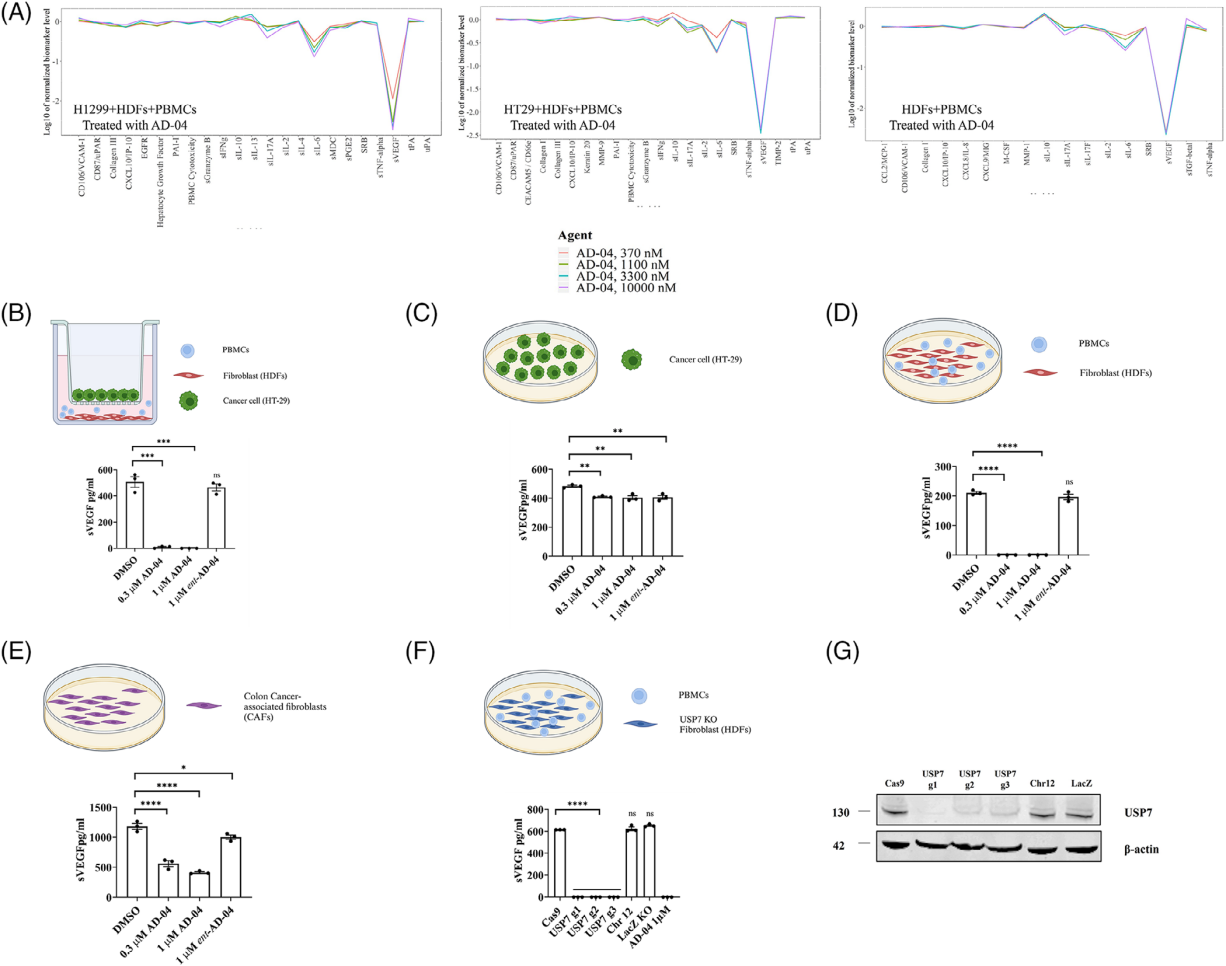
AD‐04 decreases secreted VEGF levels in activated primary fibroblasts and CAFs. (A) Co‐cultures of primary human fibroblasts with non‐small lung cancer cells (HT‐1299) or colorectal cancer cells (HT‐29) and primary human dermal fibroblasts (HDF) with activated PBMCs were treated with vehicle (DMSO) and various concentrations of AD‐04 for 48 h. Biomarker levels were measured using ELISA and presented as log‐transformed ratio. Individual cell lines or cultures were treated with AD‐04 at either .3 µm or 1 µm ent‐AD‐04 for 48 h and secreted VEGF levels were measured by ELISA in cell culture supernatants using: (B) HT‐29 cells were co‐cultured with primary human dermal fibroblasts (HDFs) and anti‐CD3/CD28‐stimulated PBMCs. (C) HT‐29 alone. (D) HDFs co‐cultured with anti‐CD3/CD28‐stimulated PBMCs, and (E) primary adenocarcinoma‐associated fibroblasts (CAFs). (F) Secreted VEGF was measured by ELISA in supernatants from USP7 CRISPR/Cas9 knock‐out HDFs co‐cultured with activated PBMCs. (G) Knock‐out efficiency was confirmed by immuno‐blot; Cas9, chromosome 12 safe region knock‐out and LacZ were used as negative controls. Data presented as mean ± S.E.M., **p < .01, ***p < .001, ****p < .0001 (ANOVA), ns not significant.

USP7 promotes the secretion of VEGF by activated fibroblasts. We used our previously described highly potent and selective USP7 inhibitor AD‐04^24^ to explore the role of USP7 in modulating the TME. AD‐04 treatment caused a dramatic > 300‐fold decrease in secreted VEGF (sVEGF) in co‐culture systems containing primary human dermal fibroblasts (HDFs) co‐cultured with stimulated PBMCs in the presence or absence of human lung (H1299) or colorectal (HT‐29) cancer cells (Figure 1A). VEGF modulation in response to USP7 inhibition was confirmed in HT‐29 cells co‐cultured with activated PBMCs and multiple primary human fibroblasts, including HDFs and primary lung fibroblasts WI‐38; whereas ent‐AD‐04, the inactive enantiomer of AD‐04,^24^ had no significant effect on sVEGF levels (Figure 1B, and Figure S1A). To confirm which cell type drives the sVEGF reduction observed in co‐cultures, we monitored VEGF secretion in each cell type independently. In cancer cells (HT‐29), AD‐04 had a minimal impact on sVEGF levels (Figure 1C). In contrast, AD‐04 markedly decreased sVEGF levels in multiple primary fibroblasts activated in a co‐culture with anti‐CD3/anti‐CD28 stimulated PBMCs (Figure 1D).^25^ The decrease in sVEGF from fibroblasts was independent of the method used to activate them as similar results were obtained following activation by FGF‐2 or TGF‐β (Figure S1B,C). Moreover, comparable decreases in sVEGF were observed in both primary colorectal adenocarcinoma fibroblasts (CAFs) (Figure 1E and Figure S1D) and lung CAFs (Figure S1D) upon AD‐04 treatment. In addition to decreasing sVEGF levels in the culture supernatant, treatment with AD‐04 also decreased levels of intracellular VEGF protein in activated fibroblasts and CAFs, but not in cancer cells. The on‐target specificity of AD‐04 in its modulation of VEGF secretion in primary human fibroblasts was further confirmed by USP7 CRISPR/Cas9 knock‐out, which resulted in complete abrogation of sVEGF in co‐cultures of HDFs with activated PBMCs (Figure 1F). The lack of impact of USP7 inhibition on sVEGF in epithelial cancer cells was not due to a lack of target engagement as AD‐04 demonstrated potent target engagement in both primary fibroblasts and cancer cells (assessed using ubiquitin activity‐based probe competition assays^26^; Figure S2). Taken together, these results demonstrate that USP7 inhibition modulates not only secreted VEGF protein but also cellular VEGF levels in activated fibroblasts.

**FIGURE 2:**
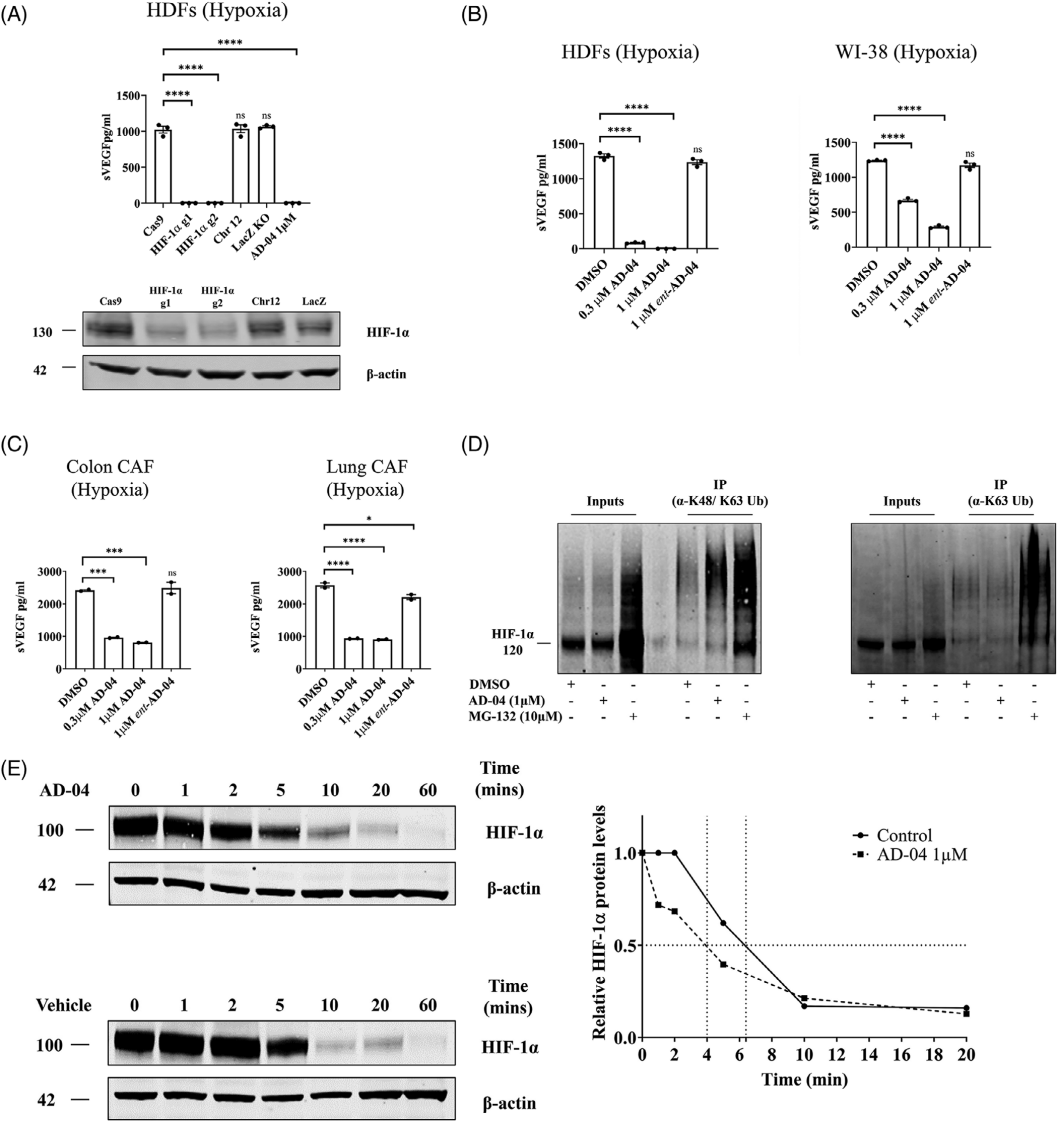
Inhibition of VEGF secretion from fibroblasts by USP7 is HIF‐1 dependent. (A) HIF‐1α CRISPR/Cas9 knock‐out HDFs were cultured under hypoxia and cell culture supernatants collected to measure secreted VEGF levels by ELISA. Knock‐out efficiency was confirmed by immuno‐blot where Cas9, chromosome 12 safe region and LacZ knock‐out were used as negative control. (B) HDFs and WI‐38 fibroblasts cultured in hypoxia and treated with AD‐04 at either .3 or 1 µm ent‐AD‐04 at 1 µm and secreted VEGF levels were measured by ELISA in cell culture supernatants. (C) Primary patient‐derived colon and lung CAF cells were cultured under hypoxia and treated with AD‐04 at either .3 or 1 µm ent‐AD‐04 at 1 µm and secreted VEGF levels were measured by ELISA in cell culture supernatants treated with AD‐04 in hypoxia. (D) Tandem Ubiquitin Binding Element (TUBE) pull‐down assay was completed in HDFs under hypoxia using either K48/K63 or K63 specific linkage Ubiquitin antibodies. Treatment with AD‐04 demonstrates increased K48/K63‐linked but not K63 HIF‐1α polyubiquitination in HDFs. (E) Cycloheximide AD‐04 reduces HIF‐1α half‐life in HDFs. Data presented as mean ± S.E.M., *p < .05, ****p < .0001 (ANOVA), ns not significant.

USP7 promotes HIF‐1α stabilization in hypoxic fibroblasts. HIF‐1α is a key modulator of VEGF expression.^7^ Therefore, as expected, under hypoxia (.1% O_2_), HIF‐1α CRISPR/Cas9 knock‐out resulted in complete abrogation of sVEGF levels demonstrating the strict requirement of HIF‐1α for VEGF expression in fibroblasts under low oxygen levels (Figure 2A). Importantly, hypoxia‐induced secretion of VEGF was inhibited by AD‐04 in HDFs and WI‐38 fibroblasts (Figure 2B) as well as in lung and colon CAFs (Figure 2C). Since HIF‐1α stability is regulated by ubiquitination^27^ and USP7 is a deubiquitinating enzyme, we investigated whether USP7 directly modulates HIF‐1α polyubiquitination. In HDFs cultured under hypoxia, polyubiquitinated proteins were pulled down using Tandem Ubiquitin Binding Entities (TUBEs, K48/K63 or K63). AD‐04 treatment increased K48 polyubiquitinated HIF‐1α accumulation (Figure 2D left panel) but not K63 polyubiquitinated species (Figure 2D right panel). Moreover, the HIF‐1α half‐life was reduced by approximately 40% in response to AD‐04 (Figure 2E). These findings indicate that USP7 inhibition increases K48 polyubiquitination of HIF‐1α and consequently accelerates its proteasome‐mediated degradation.

**FIGURE 3:**
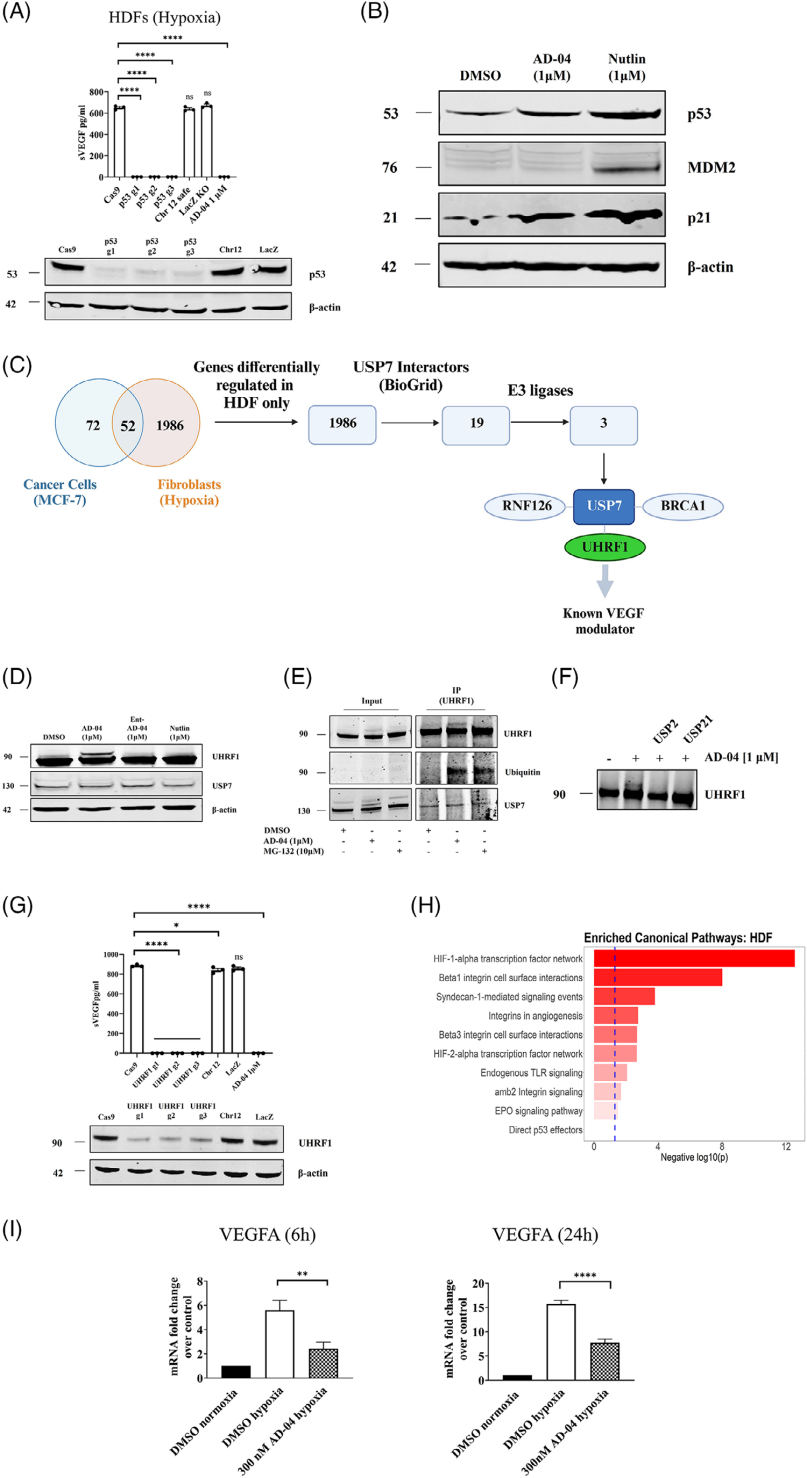
AD‐04 modulates HIF‐1α signaling pathway in hypoxia activated fibroblasts. (A) TP53 CRISPR/Cas9 knock‐out HDF cells were cultured under hypoxia and secreted VEGF levels were measured by ELISA in supernatants. TP53 knock‐out efficiency was confirmed by immuno‐blot. (B) HDF cells were treated under hypoxia for 6 h with either 1 µm AD‐04 or 1 µm Nutlin‐3a. (C) Schematic representation of genes differentially modulated by AD‐04 in activated HDFs but not in p53‐wild‐type cancer cells. Of these 2212 genes, known USP7 interactors were identified. (D) HDF cells were treated with either 1 µm AD‐04, ent‐AD‐04 or Nutlin‐3a for 6 h and immunoblotted for UHRF1. A band correlating with 8 kDa increase in UHRF1 protein size correlated with ubiquitinylation was present in AD‐04‐treated HDFs but not in MDM2 antagonist (Nutlin)‐treated cells. (E) Co‐immunoprecipitation of UHRF1 and immunoblotting with UHRF1, USP7 and Ubiquitin in HDF cells. HDFs were treated with under hypoxia for 3 h followed by 1 µm, AD‐04, 10 µm MG‐132 or DMSO. (F) Co‐immunoprecipitation of UHRF1 and immunoblotting with UHRF1 in HDF cells transfected with siRNA towards USP2 or USP21. HDF cells were treated with either DMSO or 1 µm AD‐04 for 4 h. (G) UHRF1 knock‐out HDF cells cultured under hypoxia and secreted VEGF levels were measured by ELISA in supernatants. UHRF1 knock‐out efficiency was confirmed by immuno‐blot. (H) Network pathway ranking for genes differentially regulated by AD‐04 in HDFs under normoxia versus hypoxia; AD‐04 modulates the HIF‐1α mRNA expression network (KEGG definition) in hypoxia‐activated fibroblasts. (I) HDF cells were treated with 300 nm AD‐04 under hypoxia for 6 and 24 h, VEGF mRNA expression levels were measured by RT‐qPCR.

USP7 regulates HIF‐1α in a p53‐dependent manner and deubiquitinates UHRF1. To evaluate whether USP7‐mediated modulation of sVEGF in primary fibroblasts involves its canonical target MDM2, AD‐04 was benchmarked against well characterized MDM2 antagonists. In contrast to AD‐04, MDM2 antagonists (SAR405838) did not affect sVEGF neither in activated fibroblasts nor cancer cells (Figure S3A). Notably however, while MDM2 is not involved, CRISPR/Cas9 knock‐out of p53 resulted in complete abrogation of sVEGF induction in HDFs co‐cultured with activated PBMCs (Figure 3A), suggesting that USP7 modulates VEGF expression in a p53‐dependent manner in fibroblasts. Moreover, as observed in cancer cells,^24^ inhibition of USP7 in fibroblasts leads to p53 stabilization and increased expression of its canonical target, p21 (Figure 3B) and this may offer an additional anti‐cancer benefit. MDM2 levels are very low/undetectable at baseline in fibroblasts, although they were induced in response to Nutlin.

To further explore the USP7‐dependent, MDM2‐independent mechanism of action in fibroblasts, we performed a differential gene expression analysis in cancer cells versus fibroblasts. Genes differentially regulated in response to USP7 inhibition only in fibroblasts were prioritized and overlapped with known USP7 binders. UHRF1 (Ubiquitin‐like PHD and RING finger domain‐containing protein 1) is a critical regulator of the epigenome^28^ and was the only differentially expressed gene to be a known USP7 binder as well as previously described as a VEGF modulator^29^ (Figure 3C). UHRF1 was therefore further investigated as a potential modulator of VEGF in fibroblasts. Notably, a slower migrating UHRF1 species was selectively observed in HDFs treated with AD‐04 (Figure 3D); this suggested mono‐ubiquitylation of UHRF1 in response to USP7 inhibition. Moreover, when immunoprecipitated UHRF1 proteins were probed with an anti‐ubiquitin antibody, a band was detected only in the AD‐04 treated group at the expected size for Ub‐UHRF1 (Figure 3E). USP7 was also co‐immunoprecipitated with UHRF1 indicative of their presence in a co‐complex (Figure 3E). The slower migrating band was confirmed as ubiquitylated UHRF1 when immunoprecipitated UHFR1 proteins were incubated with non‐selective recombinant catalytic domain of USP2 or USP21 post pull‐down, resulting in loss of the AD‐04‐induced slower migrating band (Figure 3F). Finally, sVEGF was not detected in supernatants from hypoxic HDFs in which UHRF1 was knocked out (Figure 3G), demonstrating its direct role in modulation of VEGF in HDFs.

To have a better understanding of USP7‐mediated VEGF modulation, a transcriptomic analysis was performed in HDFs. Gene expression was monitored by RNAseq in HDFs treated with AD‐04 under both normoxic and hypoxic conditions. A comparative pathway analysis at baseline showed that the HIF‐1α expression network was the most significantly enriched pathway in hypoxia‐activated fibroblast samples (Figure 3H). Differential induction of VEGF mRNA in AD‐04 versus control‐treated hypoxic fibroblasts was subsequently confirmed by Q‐PCR (Figure 3I). Altogether, these data demonstrate that USP7 regulates VEGF expression in activated fibroblasts at the transcriptional level by regulating the ubiquitination status of HIF‐1α and UHRF1.

**FIGURE 4:**
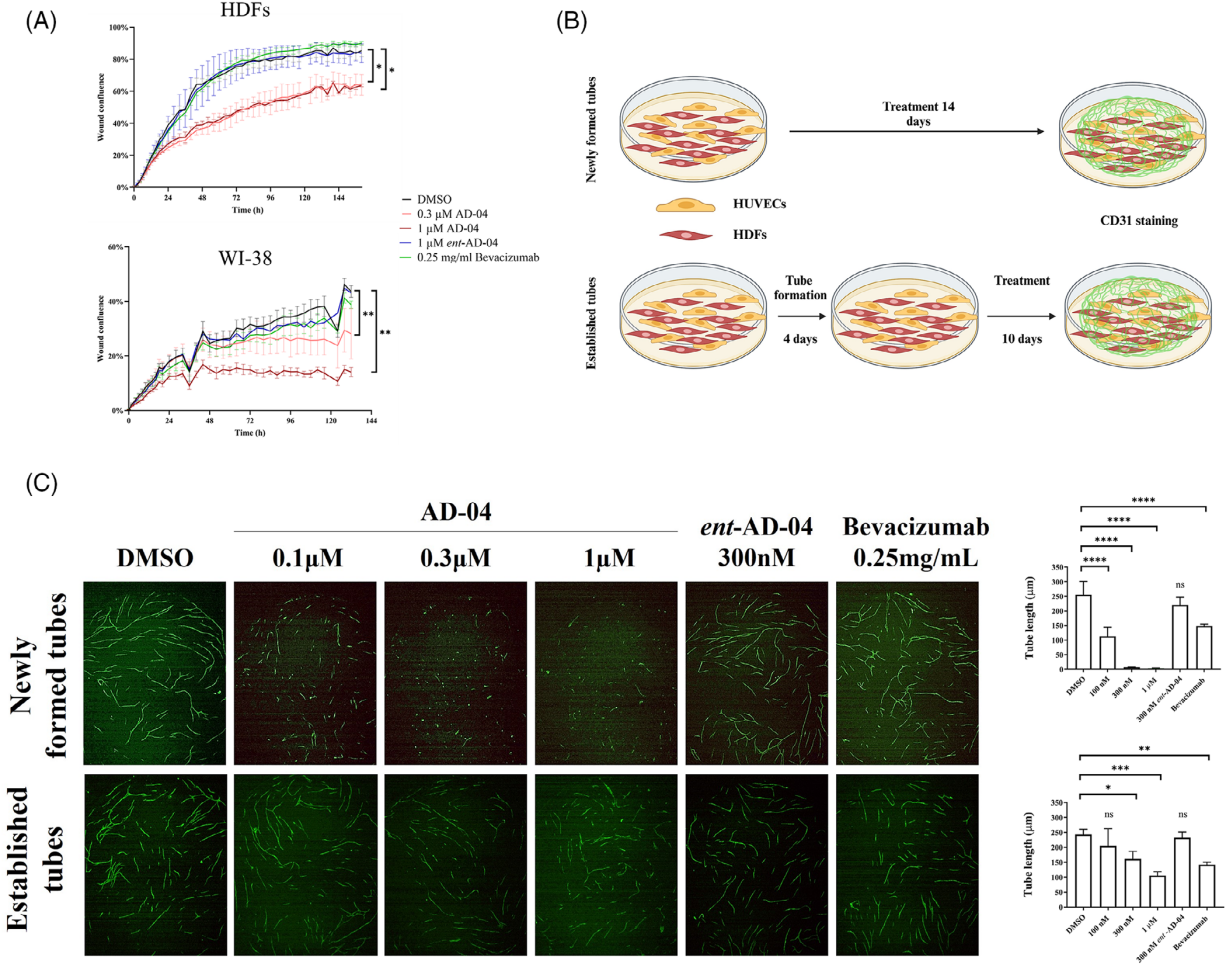
USP7 modulates cell migration and neovascularization. (A) Scratch wound invasion assays were used to analyze HDF and WI‐38 cells were with either DMSO, .3, or 1 µm of AD‐04, 1 µm ent‐AD‐04 or .25 mg/mL of VEGF inhibitor bevacizumab for 6 days. (B) Schematic representation of tubule formation experiment. (C) HDF and HUVEC co‐cultures were treated with vehicle (DMSO) and indicated concentrations of AD‐04, ent‐AD‐04 or bevacizumab prior (top) or after (bottom) tube formation then analyzed for CD31 staining. Representative pictures taken at day 14. Tubule formation was quantified by measuring tube length in microns (*right*). Data presented as mean ± S.E.M., *p < .05, **p < .01, ***p < .001, ****p < .0001 (ANOVA), ns not significant.

USP7 promotes cell invasion and blood vessel formation in vitro. To understand the functional implications of USP7‐mediated modulation of VEGF in the TME, we examined the effect of AD‐04 on proliferation, migration and invasion of the various cellular components of the tumour using live cell time‐lapse imaging. Firstly, AD‐04 had no effect on cancer cell, fibroblast, or endothelial cell proliferation (Figure S3B). Next, we examined the effect of AD‐04 treatment on migration of cells using the scratch wound cell migration assay. Again, USP7 inhibition did not impact cell migration in either cancer cells, fibroblasts or endothelial cells (Figure S3C). Finally, the impact of AD‐04 on the invasive capability of the cells was examined in a scratch wound cell invasion assay. AD‐04 showed no effect on cancer cell (CT‐26) or endothelial cell (HUVEC) invasion (Figure S3D). Activated fibroblast invasion was significantly attenuated upon AD‐04 treatment in HDFs and WI‐38 fibroblasts, but not by treatment with the anti‐VEGF antibody bevacizumab (Figure 4A). These findings demonstrate the ability of AD‐04 to specifically inhibit the invasive capacity of activated fibroblasts, distinct from anti‐VEGF agents.

In addition to their primary role in the synthesis and maintenance of the extracellular matrix (ECM), fibroblasts have the capacity to alter the mechanical extracellular microenvironment and therefore regulate vascularization processes.^30^ Fibroblast‐derived VEGF can induce, support and modulate endothelial cell sprouting and the expansion of capillary‐like networks (tubes) in vitro.^31^, ^32^ The effect of AD‐04 on the capacity of HDFs to support the formation of capillary‐like structures was therefore assessed. Fibroblast co‐cultures with primary human endothelial cells (HUVEC) were treated with AD‐04 either immediately post‐seeding or several days after seeding once tubes had already formed (Figure 4B). After 14 days, co‐culture of HDFs and HUVECs resulted in the formation of vascular tubes. Unlike bevacizumab, which only caused tube ‘normalization’, AD‐04 led to concentration‐dependent inhibition of tube formation (Figure 4C, top panel). Interestingly, when pre‐formed capillary‐like networks were treated with AD‐04, the effect on tube length was significantly less pronounced (Figure 4C, bottom panel). Taken together, these results indicate that USP7 inhibition results in inhibition of *de novo* tube formation by sVEGF modulation in fibroblasts.

**FIGURE 5:**
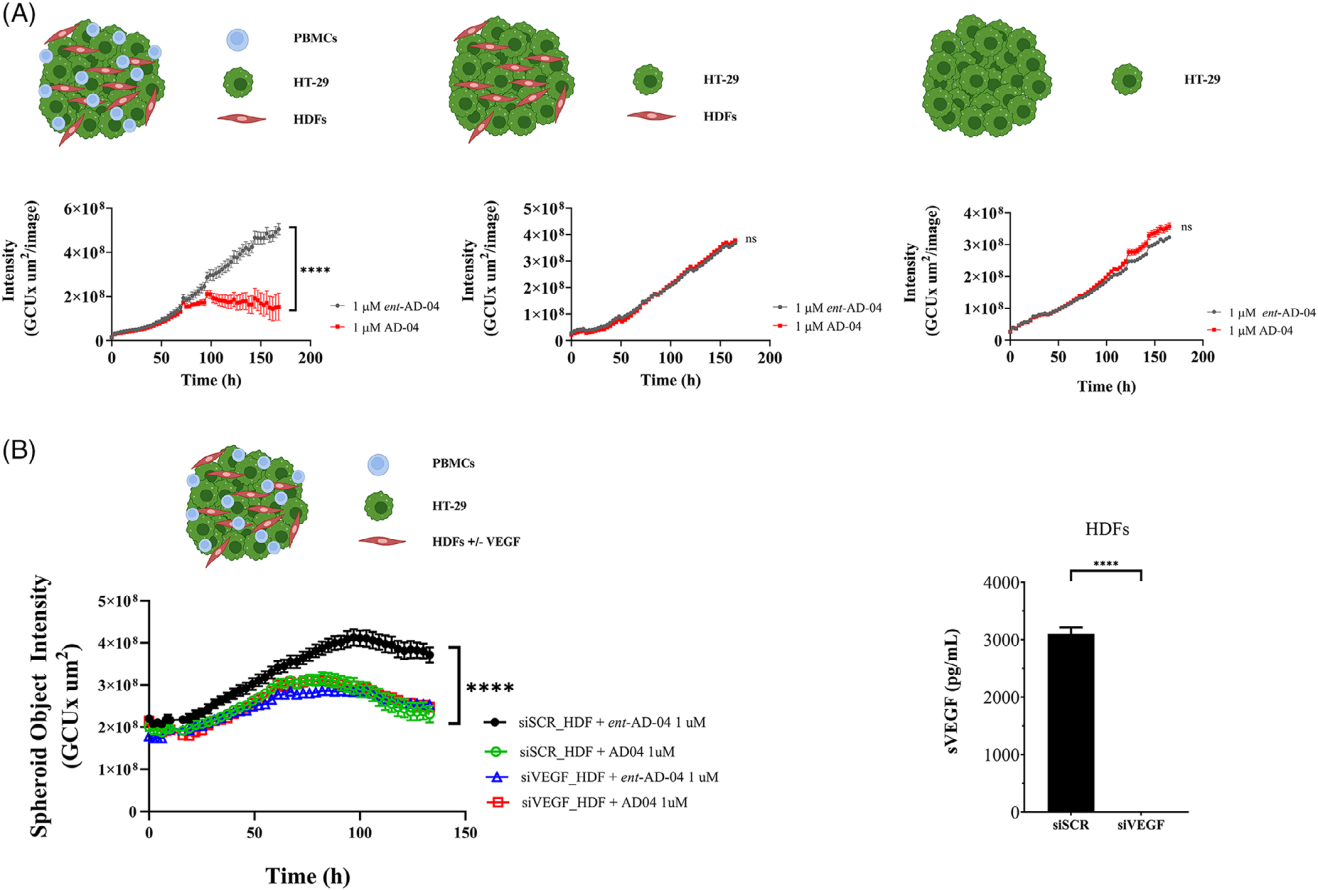
USP7‐dependent fibroblast‐derived VEGF promotes tumour cell growth and survival in co‐cultures of tumour spheroids. (A) Spheroid growth was measured over 6 days from co‐culture of HT‐29 cells, HDFs and PBMCs (top left), HT‐29 cells and HDFs (middle) or HT‐29 cells alone (top right) treated with 1 µm of either AD‐04 or ent‐AD‐04. (B) Spheroid growth was measured from HT‐29 cells, PBMCs, and siVEGF knock‐down treated HDF cells treated with either 1 µm of AD‐04 or ent‐AD‐04 for 6 days; knock‐down efficiency of siVEGF HDFs was measured by secreted VEGF ELISA (right panel). Data presented as mean ± S.E.M., *p < .05, **p < .01, ***p < .001, ****p < .0001 (ANOVA), ns not significant.

USP7‐dependent fibroblast‐derived VEGF promotes tumour cell growth and survival. To evaluate the role of USP7 on cancer cell growth under conditions where cancer cells are interacting with other TME components, we assessed the response of 3D tumour spheroids composed of cancer cells, fibroblasts and CD3/CD28‐activated immune cells to treatment with the USP7 inhibitor. AD‐04 significantly decreased the growth of spheroids formed from HT‐29 cells, primary HDFs and activated PBMCs, while ent‐AD‐04 did not have any significant effect (Figure 5A). Notably, AD‐04 had no impact on the growth of HT‐29 spheroid monocultures. The same was observed in spheroids formed from HT‐29, primary colon CAFs and activated PBMCs (Figure S4). To determine the role of fibroblast‐derived VEGF in mediating these effects, VEGF expression in HDFs was targeted using RNA interference (Figure 5B). Notably, depleting VEGF in fibroblasts phenocopied the effects of USP7 inhibition on spheroid growth in the tri‐cultures, and there was no additional effect of inhibiting USP7 in VEGF‐depleted fibroblast tri‐cultures, demonstrating the key role of fibroblast‐secreted VEGF in determining the response of tumour spheroids to USP7.

**FIGURE 6:**
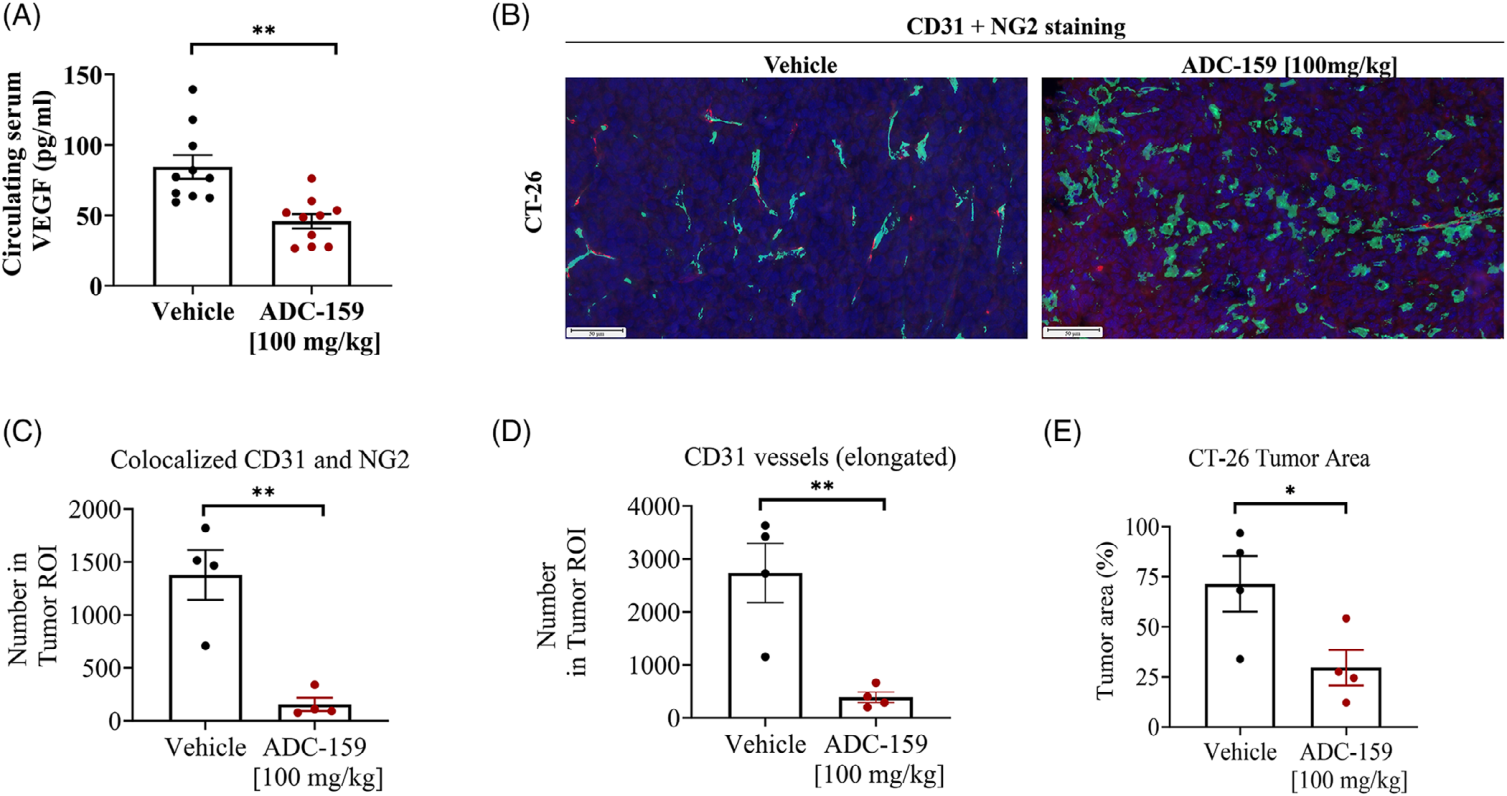
Modulation of serum VEGF and neovascularization in vivo in CT‐26 tumours in response to USP7 inhibition. (A) ADC‐159 modulated circulating VEGF levels in vivo. Serum samples from CT‐26 tumour‐bearing mice were collected after 14 daily doses and circulating serum VEGF levels measured by ELISA (*n* = 10 mice/group). Data presented as mean ± S.E.M., **p < .01 (ANOVA). (B) ADC‐159 prevented tumour vessel maturation. CT‐26 tumour FFPE blocks (*n* = 4/group) were prepared after 14 days of ADC‐159 treatment and co‐stained with CD31 (green), NG2 (red), and DAPI (blue) showing distinct changes in CD31+ vessels (green) and NG2 pericytes (red); magnification = ×10 (NPD.view2), scale bar = 50 microns. Quantitative immunofluorescence was performed to assess these changes: (C) pericyte coverage (colocalization of CD31 and NG2), and (D) elongated vessels (CD31+); ROI = region of interest. (E) Viable tumour areas were also quantified histologically.

USP7 modulates in vivo TME by decreasing VEGF, tumour vasculature and growth. In vitro data presented above indicates that USP7 inhibition has a marked effect on fibroblast‐mediated angiogenesis and impacts tumour spheroid growth, and only in the presence of fibroblasts and activated immune cells. The full anti‐tumour effects of USP7 inhibition can therefore only be assessed in an immune‐competent setting in vivo, such as in a syngeneic cancer model. Subsequently, we developed an orally bioavailable, potent and specific USP7 inhibitor, ADC‐159 (Figure S5; Table S1–S3). ADC‐159 was dosed orally to CT‐26‐bearing mice for 14 days and was very well tolerated (Figure S6A). Circulating serum VEGF levels were decreased by 46% (*p* = .001) in the ADC‐159‐treated group (Figure 6A), consistent with the modulation of VEGF observed in vitro in response to USP7 inhibition. Importantly, ADC‐159 inhibited the formation of mature blood vessels in the CT‐26 tumours (Figure 6B) as monitored by the disappearance of elongated endothelial cell structures (detected by CD31 staining). Furthermore, the co‐localization of CD31 structures with NG2, the pericyte marker of mature blood vessels was reduced by 89% (*p* = .002) in the ADC‐159‐treated group (Figure 6C). Unbiased quantification of the number of mature blood vessel within tumours (CD31+) demonstrated an 86% decrease upon ADC‐159 dosing (*p* = .0062, Figure 6D). Necrotic areas inside the tumour were significantly increased in the ADC‐159 treated group (Figure S6B). VEGF has been shown to impair leukocyte‐endothelial interactions by reducing the adhesion molecules, ICAM‐1, VCAM‐1, and LFA‐1, in angiogenic vessels and hampering the infiltration of T‐effector cells into tumours.^33^, ^34^ Notably, we found that USP7 inhibition increased gene expression levels of ICAM‐1 (log2 fold changes = .85, padj = .017), VCAM‐1 (log2 fold changes = .72, padj = 1.9 × 10^−4^), and ITGB‐2 (log2 fold changes = 1.26, padj = 1.84 × 10^−4^) in the tumour (Figure S6C). ADC‐159 treatment led to a 58% decrease in CT‐26 tumour area by histological evaluation (*p* = .044, Figure 6E). USP7 inhibition in the CT‐26 tumour model in vivo was verified by monitoring USP7 target engagement (Figure S6D) as well as by monitoring induction of the well‐characterized biomarker of p53 pathway activation, GDF‐15^35^ (Figure S6E).

**FIGURE 7:**
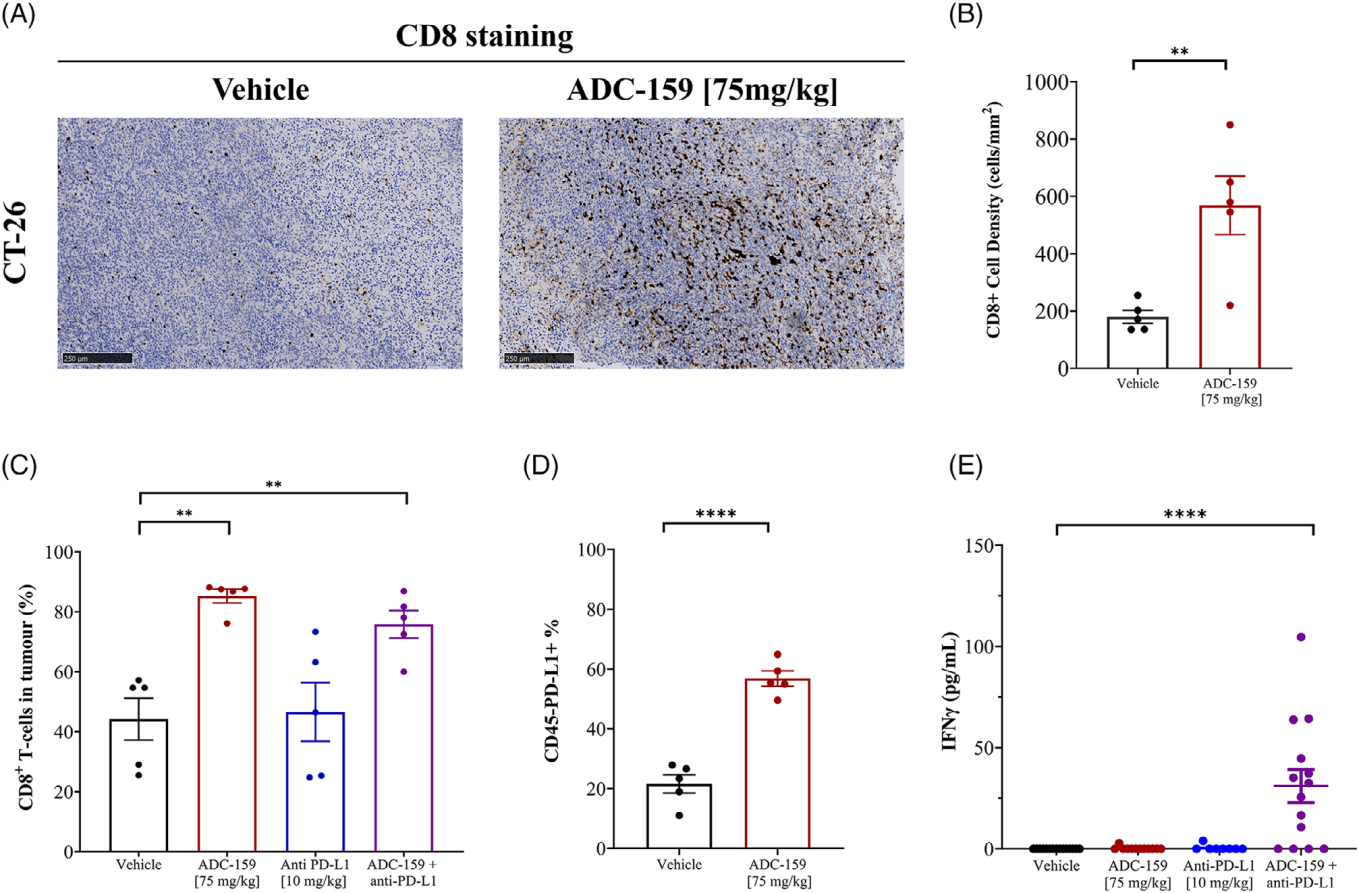
USP7 inhibition by ADC‐159 modulates the immune tumour microenvironment in CT‐26 tumours. Treatment with ADC‐159 increased (A) recruitment of CD8‐positive lymphocytes in engrafted CT‐26 tumours (*n* = 5 satellite mice/group) after 13 days of dosing as assessed by immunohistochemistry (IHC; ×10 magnification (NDP.view2), scale bar = 250 microns) and (B) quantitative IHC (data (*n* = 5) presented as mean ± S.E.M., **p < .01, ****p < .0001, unpaired *t*‐test) and (C) flow cytometry (data (*n* = 5) presented as mean ± S.E.M., **p < .01, one‐way ANOVA with Dunnett's multiple comparison test); and increased (D) PD‐L1 expression in CT‐26 tumours as measured by flow cytometry (data (*n* = 5) presented as mean ± S.E.M., ****p < .0001, unpaired *t*‐test), and increased (E) circulating serum IFNγ levels when measured by ELISA, but only when dosed in combination with anti‐PD‐L1 (data presented as mean ± S.E.M., *n* = 14 mice/group, ****p < .0001, one‐way ANOVA Brown‐Forsythe).

USP7 inhibition promotes anti‐tumour immunity leading to synergy with immune‐checkpoint‐inhibitors, improved tumour efficacy and significant gains in survival benefit. As well as its role in promoting tumour neoangiogenesis, VEGF is also known to play a role in tumour‐induced immunosuppression, including inhibiting T‐cell recruitment.^36^ It was therefore notable that ADC‐159 treatment caused a 3.16‐fold increase in CD8‐positive T‐lymphocytes per square millimetre of CT‐26 tumour as assessed by quantitative IHC (*p* = .0058, Figure 7A,B) and confirmed by flow cytometry (1.92‐fold increase, *p* = .0011, Figure 7C). Anti‐PD‐L1 treatment alone did not significantly increase the infiltration of CD8‐positive T‐lymphocytes into CT‐26 tumours, nor did it further enhance the increase observed in response to ADC‐159 (Figure 7C). Importantly, no significant effects were observed on CD4‐positive T‐helper cells or FOXP3‐positive regulatory T‐cells (Figure S7A,B). Interestingly, we observed a 2.63‐fold increase in PD‐L1 protein levels in ADC‐159‐treated tumours compared to vehicle as measured by flow‐cytometry (*p* < .0001, Figure 7D) and IHC H‐scores (16%, *p* = .03, Figure S7C,D). When assessed by Q‐PCR, we also observed a 4.56‐fold increase in CTLA‐4 levels compared to vehicle treated tumours. As a measurement of anti‐tumour immune responses we investigated IFNγ levels. At the time‐point used (13 days), only a minimal impact was observed on tumour levels of IFNγ following treatment with ADC‐159 alone. However, we observed a synergistic increase in serum IFNγ in the combination group (Figure 7F) consistent with the presence of cytotoxic anti‐tumour T‐cells in these allografted CT‐26 tumours. No changes were detected in other pro‐inflammatory cytokines such as IL‐2, which supports our early BioMAP results.

**FIGURE 8:**
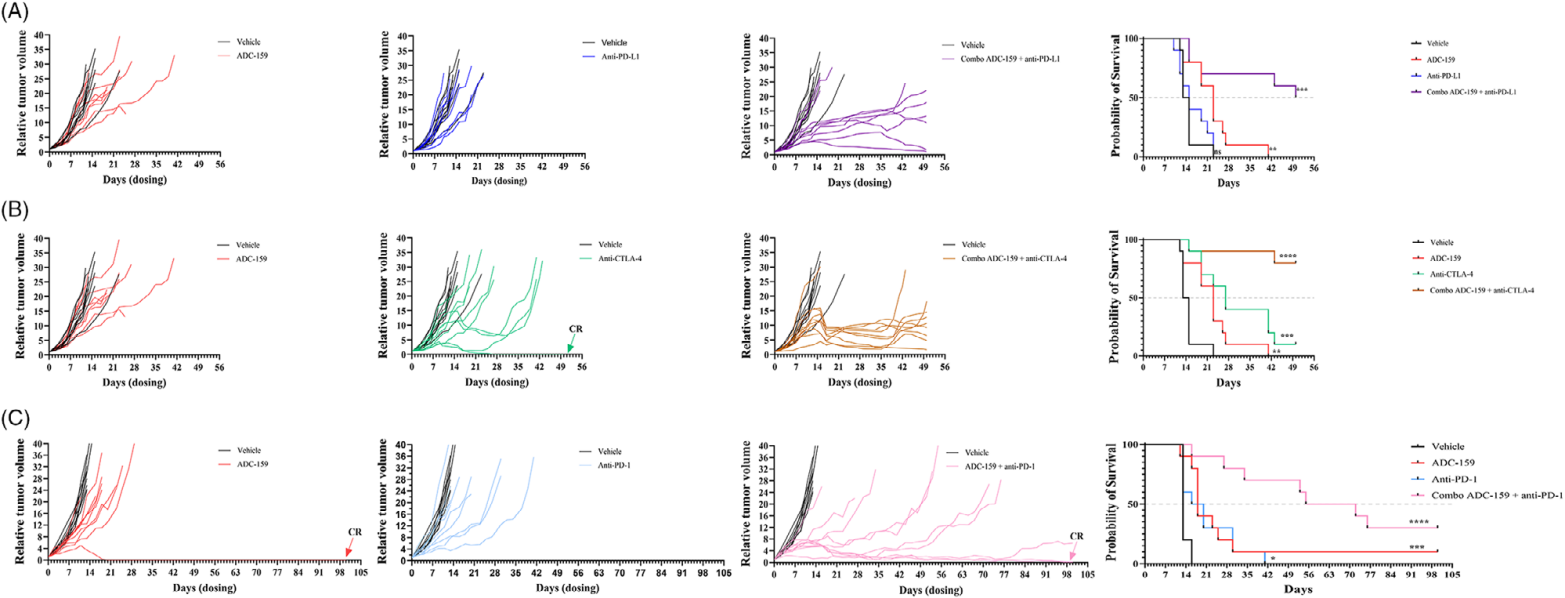
USP7 inhibition promotes anti‐tumour immunity leading to synergy with immune checkpoint inhibitors. Individual tumour response curves of CT‐26 allograft tumours (*n* = 10 mice mice/group) treated with single agent ADC‐159 and a series of immune checkpoint inhibitors, alone and in combination, leading to survival benefit. (A) CT‐26 tumours treated with vehicle (black lines), ADC‐159 alone (red lines), mouse anti‐PD‐L1 alone (dark blue lines), or ADC‐159 in combination with anti‐PD‐L1 (purple lines); Kaplan‐Meier Survival analysis of preceding treatment groups. (B) The same CT‐26 allograft study treated with vehicle (black lines), ADC‐159 alone (red lines) [included for side‐by‐side comparison], anti‐CTLA‐4 alone (green lines), or ADC‐159 in combination with anti‐CTLA‐4 (brown lines); Kaplan‐Meier Survival analysis of preceding treatment groups. (C) An independent CT‐26 allograft study treated with vehicle (black lines), ADC‐159 alone (red lines), mouse anti‐PD‐1 alone (pale blue lines), or ADC‐159 in combination with anti‐PD‐1 (pink lines); Kaplan‐Meier Survival analysis of preceding treatment groups. In all experiments, ADC‐159 was dosed once daily p.o. at 75 mg/kg and ICI agents were dosed i.p. at 10 mg/kg twice weekly (panels A and B) or every four days (Q4D; panel C) for the duration of the experiment. CR = complete responder (tumour volume = 0 for three consecutive measurements or 10 days; also classed as a tumour‐free survivor if tumour volume = 0 at study end). Kaplan‐Meier Survival curves showing improved survival benefit of ICI agents when dosed in combination with the USP7 inhibitor, ADC‐159. Survival proportions were calculated from surrogate humane tumour volume endpoints; mice surviving to study end where censored. Dotted line indicates the 50% survival fraction. Levels of statistical significance (log‐rank test), * *p* < .05, **p < .01, ***p < .001, ****p < .0001, ns not significant, *n* = 10 mice/group.

Considering these results, we went on to evaluate the functional impact of USP7 inhibition on immune checkpoint response in vivo in a series of CT‐26 tumour models. ADC‐159 was combined with anti‐PD‐L1, anti‐PD‐1 or anti‐CTLA‐4 antibodies and used to treat immune‐competent mice bearing CT‐26 allograft tumours. Importantly, combination of ADC‐159 with each of all three ICIs produced strong anti‐tumour efficacy, including durable tumour regressions and tumour responses, compared to the single agent treatments (Figure 8). This was reflected in significantly increased survival benefit using humane surrogate endpoints for each of the combination arms (Figure 8, right hand panels).

## 3 DISCUSSION

We have demonstrated that the inhibition of USP7 in the fibroblast compartment of the TME leads to a significant decrease in VEGF secreted by activated fibroblasts. This effect is specific to activated fibroblasts and not cancer cells, PBMCs, or endothelial cells. Non‐activated fibroblasts do not secrete VEGF, indicating that USP7 inhibition is unlikely to affect resting fibroblast physiology. Fibroblast activation can occur following stimulation with various factors including FGF‐2, TGF‐β, hypoxia, or interaction with immune cells. Regardless of the method of stimulation, treatment with USP7 inhibitor led to a significant decrease in VEGF secretion from activated fibroblasts and cancer‐associated fibroblasts.

VEGF is a key mediator of the immunosuppressive microenvironment.^37^ By modulating VEGF secretion from activated fibroblasts in the TME, USP7 impacts several key aspects of the TME: neovascularization, immune cell recruitment, invasion, and extracellular matrix remodelling. Fibroblasts are essential for neovascularization,^38^ and our results demonstrate that following USP7 inhibition, the ability of fibroblasts to support neovascularization is significantly impaired in vitro and in vivo. In fact, the functional importance of VEGF in tumour angiogenesis and immunosuppression reinforced the rationale for the development of VEGF/VEGFR targeting agents.^39^ Although VEGF therapies deliver favourable outcomes in some patients, improvements in progression‐free survival and quality of life can be brief leading to modest improvements in overall survival in most patients.^39^, ^40^, ^41^ This can be due to several escape/resistance mechanisms that allow tumours and immune cells to adapt to the loss of tumour vessels by either re‐establishing growth through neovascularization or by altering their growth behaviour without revascularization.^42^, ^43^, ^44^ For example, beyond neovascularization, VEGF can reduce T‐cell infiltration into tumours and influences the regulatory function of systemic immune cells, thereby reducing the anti‐tumour immune response. Furthermore, VEGF can cause clustering defects on the surface of endothelial cells by inhibiting lymphocyte adhesion to activated endothelial cells, and the subsequent trafficking of infiltrating T‐cells across the endothelia into the tumour.^45^, ^46^

Functionally, USP7 inhibition in the CT‐26 syngeneic mouse model led to increased recruitment of CD8+ lymphocytes to the tumour but did not affect CD4+ or regulatory T‐cells. “Immune desert” type tumour microenvironments are increasingly recognised as a major reason for lack of efficacy/resistance to ICIs or other immune‐targeted therapies and are linked to poor outcomes for patients in multiple cancers.^47^ Our data provide a rationale for combining USP7 inhibitors with ICIs in patients with immune desert TMEs. A small number of studies have previously linked USP7 to modulation of the TME, specifically to modulation of regulatory T‐cells.^48^, ^49^, ^50^ However, first‐generation USP7 inhibitors were used in these studies and the irreversible nature of their mode of action, undefined selectivity, and non‐optimized physicochemical properties makes it difficult to interpret the results obtained with these early pharmacological tools.^51^, ^52^ In our hands, ADC‐159, which is a second‐generation, potent, selective, and reversible USP7 inhibitor, does not significantly modulate the level of regulatory T‐cells in vivo, whilst significantly enhancing the recruitment of CD8+ lymphocytes.

Mechanistically, USP7 inhibition results in modulation of HIF‐1α polyubiquitination specifically in fibroblasts, accelerating its already fast turnover via proteasomal degradation in the TME. Previous findings in cancer cells have indicated that hypoxia‐induced K63‐polyubiquitinated USP7 deubiquitinates HIF‐1α causing CBP‐mediated H3K56 acetylation on the gene promoter of HIF‐1α to advance epithelial‐to‐mesenchymal transition and metastasis.^53^ Genetic and biochemical validation in cancer cells^54^, ^55^ have indicated that USP7 deubiquitinates and stabilizes the E3 ligase, MDM2. Surprisingly, MDM2 antagonists did not modulate VEGF expression in fibroblasts, suggesting a difference in the USP7 mode of action between cancer cells and fibroblasts. Our study demonstrates, for the first time, a critical requirement for the MDM2 target, p53, in modulating VEGF expression in fibroblasts. p53 has been reported to interact directly with HIF‐1α, thereby modulating VEGF expression. However, reports are conflicting,^56^, ^57^, ^58^, ^59^, ^60^ and no previous study has examined the role played by USP7 in modulation of VEGF expression in human fibroblasts.

In addition to HIF‐1α and p53, we found that the UHRF1 E3 ligase is part of the USP7‐mediated regulation of VEGF in fibroblasts. UHRF1 is a key epigenetic regulator and recruits DNA methyltransferase 1 (DNMT1) to methylated DNA.^61^ USP7 is a well‐characterized regulator of polycomb repressor complex 1 (PRC1), a critical epigenetic regulator.^62^ It has been reported that USP7 binds to UHRF1, promoting its deubiquitination thereby increasing its stability and chromatin binding.^63^ We found that USP7 inhibition resulted in the generation of a mono‐ubiquitylated form of UHRF1 in hypoxic fibroblasts and that UHRF1 was absolutely required for VEGF production in hypoxic fibroblasts. Previous studies have linked DNMT1 and UHRF1 to the modulation of VEGF signalling, but only in cancer cells.  Overall, these results suggest that USP7‐dependent, UHRF1‐mediated epigenetic regulation of the VEGFA locus is necessary to enable efficient recruitment of HIF‐1α and p53 to drive transcription in hypoxic fibroblasts.

**FIGURE 9:**
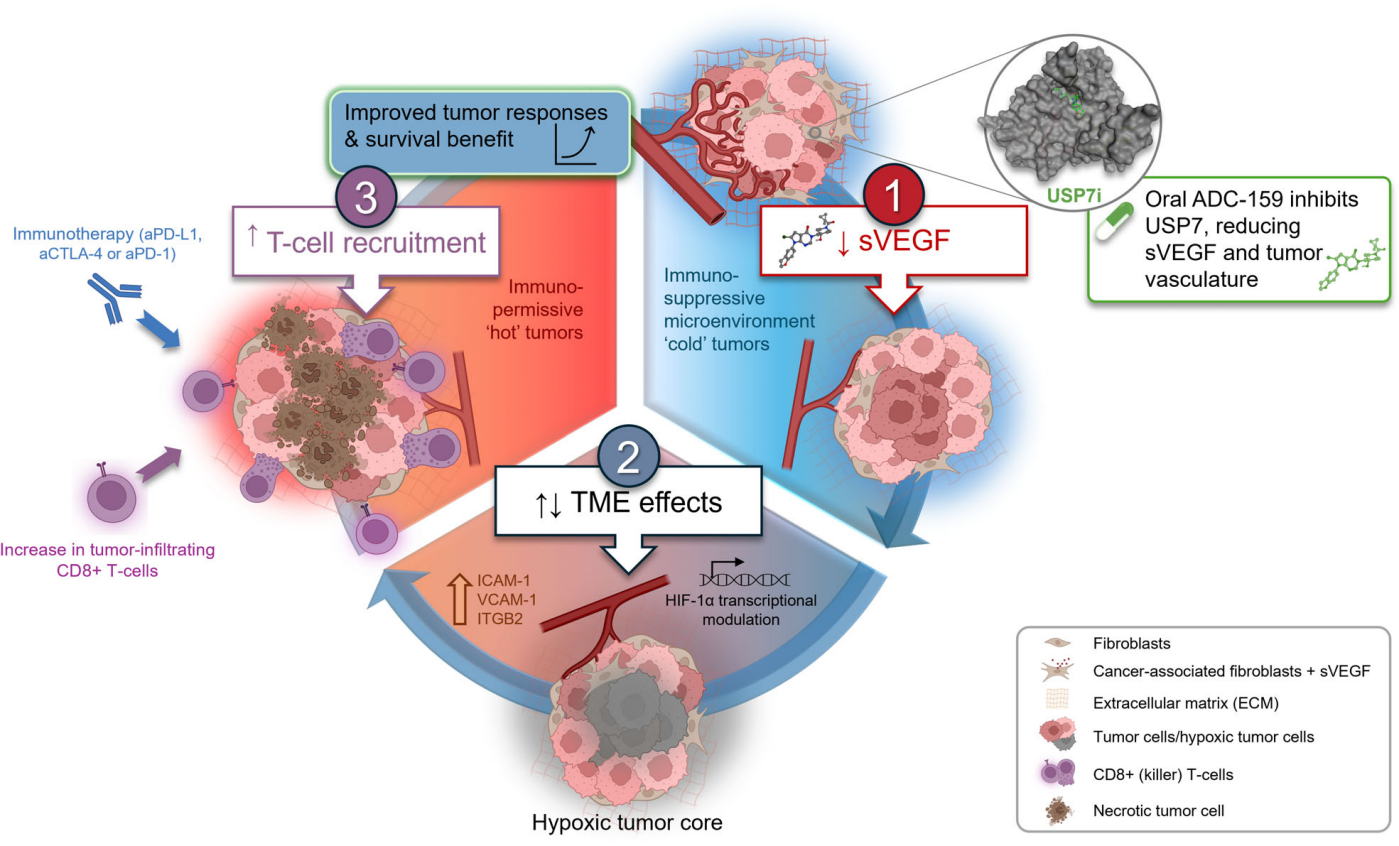
Conceptualization—Pharmacological inhibition of USP7 delivers multi‐modal effects on the tumour microenvironment which translates into benefit in combination with immunotherapies. (1) The oral USP7 inhibitor, ADC‐159, reduces sVEGF from CAFs and impacts tumour vasculature. (2) USP7 inhibition affects HIF‐1α transcriptional modulation, tumour hypoxia and remodelling of the tumour microenvironment creating a permissive immune micro‐climate for infiltrating lymphocytes—turning immunologically ‘cold’ tumours, ‘hot’. (3) In preclinical models, combination treatment of ADC‐159 with immunotherapy agents delivers improved anti‐tumour efficacy and survival.

In summary (Figure 9), we have identified a novel, therapeutically relevant mode of action of USP7 in modulating and reprograming the TME by directly impacting VEGF in fibroblasts. USP7‐mediated reprograming of the TME is not linked to its previously characterized role in modulating MDM2 but does require p53 and UHRF1 in addition to HIF‐1α. This represents a function of USP7 that is unique to primary or cancer‐associated fibroblasts, and which is not observed in cancer cells or other cells present in the TME. Given the potential for USP7 inhibitors to transform “immune desert” tumours into “immune responsive” tumours, this paves the way for a novel therapeutic strategy combining USP7 inhibitors with ICIs. As such, we propose USP7 inhibitors as a new class of small molecule immune modulators specifically targeting cancer‐associated fibroblasts with therapeutic potential in solid tumours that are unresponsive to existing immune oncology treatments.

## 4 MATERIALS AND METHODS

### 4.1 Materials and reagents

The MDM2 antagonists Nutlin‐3a (Tocris; #3984), SAR405838 (MedchemExpress; MI‐773, #HY‐17493) and RG7112 (MedchemExpress; #HY‐10959), bevacizumab (Genentech; CAS, 216974‐75‐3), pembrolizumab (Merck, Kenilworth, NJ, USA), anti‐mouse PD‐L1 (10F9G2, BioXcell, USA), anti‐mouse CTLA‐4 (9D9, BioXcell, USA), and anti‐mouse PD‐1 (RMP1‐14, BioXcell, USA) were purchased from commercial suppliers as indicated and used with no further purification. AD‐04 and ent‐AD‐04 were synthesized according to O'Dowd et al.^23^ ADC‐159 was synthesized according to the procedure outlined in the Supporting Information.

### 4.2 Cells and culture conditions

All primary cells and cell lines were obtained from the American Type Culture Collection (ATCC), authenticated by STR profiling (Promega) and shown to be mycoplasma‐free using the MycoAlert mycoplasma detection (LT07‐318; Lonza). For growth, cells were maintained at 37°C in a humidified atmosphere with 5% CO_2_. HT‐29 (colorectal) cells were cultured in McCoy's medium 5A supplemented with 10% (v/v) FBS, 1% (v/v) penicillin–streptomycin, 1% (v/v) L‐glutamine. LNCaP (prostate) cells were cultured in RPMI supplemented with 15% (v/v) FBS, 1% (v/v) penicillin‐streptomycin, 1% (v/v) l‐glutamine. H1299 (lung), and CT‐26.WT (mouse colon carcinoma) cells were cultured in RPMI supplemented with 10% (v/v) FBS and 1% (v/v) penicillin‐streptomycin. MCF7 (breast cancer; WT p53) cells were cultured in Eagle's Minimum Essential Medium supplemented with 10% (v/v) FBS, .01 mg/mL human recombinant insulin and 1% (v/v) penicillin‐streptomycin. HDF were cultured in fibroblasts basal media supplemented with Fibroblast Growth Kit–Low Serum (final concentration for each component was as follows: l‐glutamine 7.5 mM; rhFGF basic 5 ng/mL; rhInsulin 5 µg/mL; Hydrocortisone 1 µg/mL; Ascorbic acid 50 µg/mL; FBS 2%). WI‐38 were cultured in Eagle's Minimum Essential Medium supplemented with 10% (v/v) FBS. In order to acquire myofibroblast phenotype, WI‐38 and HDFs were co‐cultured with anti‐CD3/anti‐CD28 stimulated PBMCs or were stimulated by FGF‐2 or TGF‐β. HUVEC were cultured on flasks coated with .2% gelatine in vascular cell basal media supplemented with Endothelial Cell Growth Kit‐BBE (final concentration for each component is as follows: Bovine brain extract (BBE) .2%; rhEGF 5 ng/mL; l‐glutamine 10 mm; Heparin sulphate .75 Units/mL; Hydrocortisone 1 µg/mL; Ascorbic acid 50 µg/mL, and 2% FBS: 2%). Medium and supplements were purchased from Life Technologies and ATCC except where indicated. Frozen primary human PBMCs were purchased from Cambridge Biosciences and BioIVT. Cancer associated fibroblasts (CAFs, colon cancer CAF05 and lung cancer CAF07‐AD) were purchased from Vitro Biopharma. CAFs were maintained and expanded in VitroPlusIII low serum, complete medium (Vitro Biopharma, Cat. No. PC00B1).

### 4.3 Target engagement assay

HT‐29 and HDF cells were treated with vehicle (DMSO) or USP7 inhibitors for 2 h. HDF were placed in hypoxic chamber while other cells remained in normoxia. Following incubation, cells were washed extensively thrice with 1× PBS and harvested in TE lysis buffer containing 50 mm Tris–HCl (pH 7.4), 150 mm NaCl, 5 mm MgCl_2_, .5 mm EDTA, .5% NP40, 10% glycerol, 2 mm DTT, and clarified cell lysates (30 µg) incubated with Ub‐PA (8 µg/mL final concentration; UbiQ‐057 from UbiQ) in assay buffer containing 50 mm Tris–HCl (pH 7.6), 5 mm MgCl_2_, 250 mm sucrose, .5 mm EDTA, and 2 mm DTT for 30 min. The reaction was terminated by the addition of LDS sample buffer (Life Technologies) and heated to 95°C. Samples were then analyzed by western blotting using the anti‐USP7 antibody (#4833; 1:1000 dilution). EC_50_ values were determined upon densitometry analysis. Band intensities were quantified using ImageJ software where the upper bands (USP7‐Ub) and lower bands (USP7) were calculated as a percentage of the corresponding DMSO controls (±Ub‐PA) and values were then normalized to the sum of the lower and upper bands for each concentration. The same procedure was followed for ex vivo tumour samples.

### 4.4 Cell proliferation assays

Cells were seeded in 96‐well plates at a density of 3000 cells/well. Cell proliferation was monitored with IncuCyte live cell analysis imaging system (Sartorius). Data are presented as mean ± S.E.M. (*n* ≥ 3).

### 4.5 In vitro co‐culture tube formation assay and immunostaining

HDFs were seeded in 96‐well plates to form a monolayer followed by addition of HUVECs. To assay the effect on new vessel formation, cultures were treated with AD‐04 (10 nm–1 µm), ent‐AD‐04, DMSO or bevacizumab 24 h post‐seeding. The effect on existing vessels was assayed by allowing tubes to form prior to treatment. Cells were treated every 3 days during 14 days of incubation, followed by washing with PBS and fixation with 4% formaldehyde for 15 min at room temperature (RT). Cells were permeabilized with 1× PBS containing .1% Triton X‐100 for 5 min at RT and blocked 30 min in 1% BSA/PBS. Afterwards, cells were incubated with primary CD31 antibody (MA5‐13188; 1:50 dilution) followed by incubation with secondary Alexa fluor 488F (A28175; 1:2000). Immunolabelled samples were counterstained with Hoechst 33342 nuclear dye (#62249, Thermo Fisher; 1:1000 dilution). Tubes were visualized with INCell Analyzer 2000 using a 2X magnification objective. Tube length was measured and quantified using the AngioTool software for each imaging session (available in the public domain at https://ccrod.cancer.gov/confluence/display/ROB2/Downloads).

### 4.6 Ubiquitination assays

HDFs were incubated under hypoxic condition for 3 h followed by 1 h treatment with a proteasome inhibitor, MG132 at 10 µm, AD‐04 at 1 µm, or DMSO. Cells were lysed in a buffer containing 50 mm Tris‐HCl (pH 7.5), 150 mm NaCl, 1 mm EDTA, 1% NP‐40, 10% Glycerol, 50 mm NaF, 5 mm sodium pyrophosphate, 10 mm glycerol phosphate, 1 mm sodium orthovanadate, protease (PhosSTOP, Roche), and phosphatase inhibitor cocktail tablet (complete Mini, Roche), 50 µm PR‐619 (SML0430; Sigma), 10 mm *N*‐ethyl maleimide (E3876; Sigma), and 25 µm MG‐132 (SML1135; Sigma). Lysates were pre‐cleared by centrifugation at 4°C for 15 min at 16 000×*g* and protein concentration determined using BCA protein kit assay (#23227; Thermo Fisher Scientific). 20 µL of samples was taken as input. Pre‐cleared supernatant containing .5 mg of total protein was added to 20 µL equilibrated Agarose‐TUBE 1 or TUBE 2 (UM401 or UM402; Life Sensors) beads and incubated for 2 h at 4°C on a rocker platform. Beads were collected by low‐speed centrifugation (1000–5000 × *g*, 4°C) for 5 min and washed twice with 1 mL TBS‐T (20 mm Tris‐HCl, pH 8.0, .15 m NaCl, .1% Tween‐20). Finally, beads were resuspended in 30 µL of LDS sample buffer (Life Technologies) and boiled for 10 min at 95°C. Samples were subjected to Immuno‐blot analysis by using anti‐HIF‐1α antibody (#14179; 1:500 dilution).

### 4.7 In vitro deubiquitination assay with USP2 and USP21 catalytic domain

HDF cells were treated with AD‐04 and Nutlin‐3a for 4 h followed by lysis in RIPA buffer (10 mm Tris, pH 7.5, 100 mm NaCl, 1% NP‐40, .1% SDS, 1% sodium deoxycholate, and 50 mm NaF) supplemented with protease and phosphatase inhibitors, and 10 mm *N*‐ethylmaleimide (NEM). Cell lysates were cleared by centrifugation, equal amounts for each condition were incubated with prewashed protein G agarose and anti‐UHRF1 antibody (MABE308; 1:250 dilution) for 2 h at 4°C. Beads were washed three times with RIPA buffer, twice with deubiquitination assay buffer (50 mm HEPES pH 7.3, .5 mm EDTA) and finally resuspended in 250 mL of deubiquitination assay buffer with 2 mm DTT. Samples with or without USP2 catalytic domain (100 nm; R&D, E‐504‐050) or USP21 catalytic domain (100 nm; R&D, E‐622‐050) were incubated in a thermoshaker for 8 h (37°C, 1000 rpm). Beads were then washed twice with 10 mm Tris pH 7.5, proteins eluted with SDS sample buffer and samples analyzed by immunoblotting with anti‐UHRF1 antibody (#12387; 1:1000 dilution).

### 4.8 Cycloheximide chase assay

HDF cells were incubated for 3 h in hypoxic conditions followed by treatment with AD‐04 ± cycloheximide (100 µg/mL) to block nascent protein synthesis. Cells were harvested and lysed in Laemmli sample buffer (S3401; Sigma) at different time points and subjected to western blot analysis using anti‐HIF‐1α antibody (#14179; 1:500 dilution) and β‐actin (#A5316; 1:2000 dilution) as a loading control.

### 4.9 Western blotting

Samples were run on a NuPAGE 4–12% bis‐tris or tris‐acetate protein gel (ThermoFisher) and transferred to a nitrocellulose membrane, which was blocked and incubated overnight with primary antibody at 4°C, washed with TBS‐T, incubated with secondary antibody followed by washing with TBS‐T and imaged using an Odyssey CLx (Li‐Cor). Western blotting analyses were carried out using primary antibodies purchased from Cell Signaling Technology; anti‐USP7 (#4833; 1:1000 dilution), anti‐HIF‐1α antibody (#14179; 1:500 dilution), anti‐Vinculin (#13901; 1:2000 dilution), anti‐β‐actin (#8457; 1:2000 dilution), anti‐p21 (#2947, 1:1000 dilution), Sigma; anti‐β‐actin (#A5316; 1:2000), Santa Cruz; anti‐p53 (sc‐126; 1/500 dilution), Millipore; anti‐MDM2 (#OP46; 1:200 dilution). Secondary antibodies were used at 1:5000 dilution and purchased from LiCOR Biosciences. Specific antibodies used are as follows: IRDye 800CW Goat anti Mouse IgG (925‐32210), IRDye 680LT Goat anti Mouse IgG (925‐68020), IRDye 800CW Goat anti Rabbit IgG (925‐32211), and IRDye 680LT Goat anti Rabbit IgG (925‐68021).

### 4.10 Co‐immunoprecipitation assays

HDFs were treated with a proteasome inhibitor, MG132, AD‐04, or DMSO and incubated in hypoxia for 4 h. Cells were lysed as described above. 20 µL of samples was taken as input. Pre‐cleared supernatant containing .5 mg of total protein was added to 50 µL of Dynabeads Protein A (10002D; Thermo Fisher Scientific) coupled to anti‐UHRF1 antibody (MABE308; 1:250 dilution) or anti‐USP7 antibody (#4833; 1:50 dilution) and incubated with rotation for 10 min at room temperature. Beads were collected by placing tubes on magnet and washed three times with TBS‐T. Afterwards, beads were resuspended in 30 µL of LDS sample buffer (Life Technologies) and boiled for 10 min at 95°C. Samples were subjected to Western blot analysis by using anti‐ubiquitin antibody (BML‐PW8810; 1:500 dilution), anti‐USP7 antibody (#4833; 1:1000 dilution), or anti‐UHRF1 antibody (#12387; 1:1000 dilution).

### 4.11 Detection of VEGF using enzyme‐linked immunosorbent assay

For the co‐culture experiments, 5×10^4^ cancer cells were seeded in transwells in .5 mL complete growth medium and placed on the top of 1 ×10^4^ fibroblasts plated in 12‐well in 1 mL low serum growth media overnight. Next day, cancer cells were washed with PBS and media replaced with reduced (1%) serum growth media. 5×10^5^ PBMCs were plated on top of fibroblasts, incubated for 45 min and activated with CD3 (BD Bioscience; Clone HIT3a, 1 µg/mL final concentration) and CD28 (BD Bioscience; clone CD28.2, 5 µg/mL final concentration). Afterwards, co‐cultures were treated with vehicle (DMSO), AD‐04, and ent‐AD‐04 for 48 h at 37°C in 5% CO_2._ Monoculture experiments, 1×10^4^ cells were seeded in 12‐well in 1 mL of complete growth media (cancer cells) or low serum growth media (fibroblasts) and incubated overnight. Next day, cancer cells were washed with PBS and media replaced with reduced (1%) serum growth media followed by treatment with vehicle (DMSO), AD‐04, ent‐AD‐04, MDM‐2 antagonists nutlin‐3a (#3984; Tocris), SAR405838 (#HY‐17493, MI‐773; MedchemExpress), and RG7112 (#HY‐10959; MedchemExpress) for 48 h at 37°C in 5% CO_2_ in normoxia or hypoxia. Afterwards, cell culture media was collected, cell debris were removed by centrifugation, and the concentration of VEGF in the cell culture supernatants was measured using the human/mouse VEGF immunoassay Quantikine ELISA kit (R&D systems, Minneapolis, MN, USA) according to the manufacturer's instructions. For the intracellular VEGF detection, total protein concentration was determined by BCA protein kit assay (#23227; Thermo Fisher Scientific) and VEGF levels determined using the same kit described above.

### 4.12 BioMAP phenotypic screen

BioMAP primary human cell systems (Diversity plus; Eurofins, USA) were screened. These studies follow the guidelines for human subject research under HHS human subject regulations (45 CFR Part 46) for the United States. Human neonatal foreskin fibroblasts (HDFs) from three donors were pooled and cultured according to the supplier's (Lonza, Inc., Allendale, NJ) recommendation. Peripheral blood mononuclear cells (PBMCs) were prepared from buffy coats from healthy human donors according to standard methods. Autoimmune HDFSAg system consisted of primary human dermal fibroblasts (HDFs) was co‐cultured with PBMCs stimulated with Superantigens (SAg) to model chronic T‐cell activation and inflammation. Stromal oncology colorectal cancer and non‐small cell lung cancer (NSCLC) panels were composed of cancer cells (HT‐29 or H1299), HDFs and PBMCs stimulated with SAg. This model captured the interactions between tumour cells, stimulated immune cells and the host stromal network. Co‐cultures were activated with SAg, (20 ng/mL) and treated with vehicle and AD‐04 at the concentration of 10, 3.3, 1.1, and .37 µm for 48 h. Biomarkers were measured in co‐culture supernatants by ELISA: MCP‐1, VCAM‐1, Collagen I, IP‐10, MMP‐1, sIL‐10, sIL‐17A, sIL‐17F, sIL‐2, sIL‐6, SRB, sTGFβ, sTNFα, sVEGF, IL‐8, MIG, MCSF, uPAR, Col‐III, IP‐10, EGFR, HGF, Pal‐1, PBMC Cytotoxicity, tPA, uPA, sGranzymeB, sPGE2, sIFg, Sil‐13, sMDC, Collagen III, MMP‐9, TIMP‐2, CEACAM5, and Keratin 20. Biomarker levels are presented as log‐transformed ratios $\left(\log10\frac{AD-04}{\mathrm{Vehicle}\;\mathrm{control}}\right)$.

### 4.13 CRISPR/Cas9 RNPs knock‐out

USP7 specific crRNA and the non‐specific tracrRNA (both Integrated DNA Technologies‐IDT, Coralville, IA, USA) were mixed in equimolar concentrations in a microcentrifuge tube to form tracrRNA:crRNA duplex (guide RNA). Samples were heated at 95°C for 5 min and allowed to cool‐down at RT. To form a ribonucleoprotein (RNP) complex of recombinant Cas9 coupled to the guide RNA, Cas9 enzyme (IDT, 21 µm final concentration) was added to the tracRNA:crRNA duplex. RNP complex was incubated at RT for 20 min. Prior to electroporation, HDFs were harvested by trypsinization and washed with PBS. Pellets containing 5×10^5^ cells were mixed with 94 µL of Nucleofector solution (Amaxa Human Dermal Fibroblasts Nucleofector, Lonza), 5 µL of the correct RNP or 2 µg total pmaxGFP and 1 µL of Alt‐R Cas9 Electroporation Enhancer (IDT, final concentration 1 µm) were added to each tube, mixed and transferred to electroporation cuvette. Subsequently, the cells were nucleofected by using the U‐020 program from the nucleofection device (Nucleofector IIb Device, Lonza) and 500 µL of pre‐warmed culture medium was immediately added to the cells. Cells were grown for 9 days allowing the phenotype to develop. Cells were subsequently harvested and lysed in radioimmunoprecipitation (RIPA) buffer containing 50 mm Tris–HCl (pH 7.6), 150 mm NaCl, 1 mm EDTA, 1.0% NP40, .25% Na‐deoxycholate, and supplemented with a phosphatase (PhosSTOP, Roche), protease inhibitor cocktail tablet (complete Mini, Roche), 10 mm *N*‐ethyl maleimide (#692905; Sigma) and 25 µM MG‐132 (SML1135; Sigma). 30 µg of protein was used for western blot analysis. .1×10^5^ cells were plated in 12‐well in 1 mL low serum growth media under hypoxic conditions for 48 h and VEGF levels were determined in cell culture supernatants using the VEGF immunoassay Quantikine ELISA kit (R&D systems, Minneapolis, MN, USA). The full list of sgRNAs used is detailed in Table 1

**TABLE 1:** CRISPR sgRNAs.

Target gene	Guide no.	sgRNA sequence (5′−3′)
USP7	1	GTGTACATGATGCCAACCGA
USP7	2	CTACGTCGGCTTAAAGAATC
USP7	3	TGATGGACACAACACCGCGG
HIF1A	1	ACTAAAGGACAAGTCACCAC
HIF1A	2	ACTTTGTCTAGTGCTTCCAT
TP53	1	ATGTGTAACAGTTCCTGCAT
TP53	2	TCCACTCGGATAAGATGCTG
TP53	3	CACTTTTCGACATAGTGTGG
LacZ	1	GAAGTGTTGCCATTCAATTC
Chr12 safe region	1	GCTGGTGGTCAGATGCGGGA

### 4.14 Migration and invasion assays

96‐well ImageLock plates (Sartorius) were coated with .1 mg/mL growth factor reduced Matrigel (#356230; Life Sciences) and incubated for 1 h at 37°C. Cells were seeded a density of 10 000–40 000 cells/well in 100 µL/well and incubated overnight. Next day, wounds were simultaneously created in all wells using the IncuCyte WoundMaker (Sartorius). After wounding, media was aspirated from each well and cells were washed twice with PBS. For invasion cells were overlayed with 50 µL of the Matrigel top layer at 3 mg/mL and incubated for 30 min at 37°C. Afterwards, 100 µL of culture media containing vehicle (DMSO), AD‐04, ent‐AD‐04 and bevacizumab was added to each well. Cell plates were placed into the IncuCyte live‐cell analysis system (Sartorius) and each well imaged using 10× objective every 2 h for the total of 5 days. Images were analyzed using IncuCyte Cell Migration and Invasion software (Sartorius) and results presented as percentage of wound confluence.

### 4.15 CT‐26 in vivo syngeneic tumour model

CT‐26 mouse colorectal cancer cells were grown in RPMI supplemented with 10% foetal bovine serum at 37°C in an atmosphere of 5% CO_2_ in air and were mycoplasma‐free. Cells in exponential growth phase were harvested for implantation into female BALB/c mice, approximately 6−8 weeks old at initiation of the experimental phase. Each mouse was injected subcutaneously in the right rear flank with 5×10^5^ viable CT‐26 tumour cells in .1 mL of PBS. Mice were palpated and weighed twice per week until tumours were measurable with electronic callipers. Tumour volume (mm^3^) was then estimated three times a week using the equation: Tumour volume (TV) = .5((*W*)*^2^* × *L*), where *W* is the shortest tumour diameter (width) and *L* is the longest perpendicular diameter (length), in millimetres. Body weights were measured on the same days, and daily during the dosing phase. When the mean tumour volume of the implanted cohort reached approximately 75 mm,^3^ fit and healthy mice were randomized to treatment groups (*n* = 10/group) using a stratified randomization approach so that there was no statistical difference (ANOVA Brown‐Forsythe) between the group tumour volumes (StudyDirector software version 3.1.399.19, StudyLog Systems Inc., South San Francisco, USA). Group sizes were prospectively estimated using an in‐house Power Calculator (Microsoft Excel with macro functionality) and historical tumour volume data from 5 independent CT‐26 in vivo growth curves using unlogged paired data and the following powering criteria: 80% power (2‐sided), 95% probability, and a predicted effect size of 50%. An additional animal was assigned to the estimated group size to allow for technical issues without losing statistical power. The day of randomization/group assignment was denoted as Day 0 and dosing commenced the following day. Dosing and tumour/body weight measurements were conducted in a laminar flow cabinet and clinical observations were carried out at least once daily. All in vivo study data was collected in StudyDirector software version 3.1.399.19 (StudyLog Systems Inc., South San Francisco, USA). The vehicle or ADC‐159 (75 mg/kg or 100 mg/kg) was administered orally (p.o.) once daily to ensure free‐plasma coverage over the cellular TE EC_50_, while immune checkpoint inhibitors (10 mg/kg) were dosed intraperitoneally (i.p.) once every 4 days. Mice (*n* = 5) were housed in individually ventilated cages (IVCs) with ad libitum access to food and water and provided with environmental enrichment (mouse house, cylinder and tissue nesting material). Humane surrogate endpoints were applied to all groups as follows: tumour volume not to exceed 1800 mm,^3^ tumour ulceration not to exceed 25% of tumour surface area or reach full skin thickness, and body weight loss not to exceed 20% of starting weight on Day 0. These humane surrogate endpoints were applied to the Kaplan‐Meier survival analyses. Mice were euthanized at humane surrogate endpoints using approved humane methods carried out by skilled operators. All procedures involving the care and use of animals were approved by a local IACUC group and conducted by trained personnel in accordance with AAALAC regulations and good veterinary practice.

### 4.16 Flow cytometry

Harvested tumours were collected in HBSS medium, minced and incubated 30 min at 37°C in non‐enzymatic cell dissociation buffer followed by mechanical dissociation through a 70 µm filter. Viable cells were then enriched using Ficoll gradient. All cell suspensions were counted, and one million viable cells were seeded in 96‐well plates in 100 µL of staining buffer for acquisition. Non‐specific binding was performed using mouse FcR blocking reagent. Fixable Viability Dye eFluor 780 (eBiosciences, 65‐0865‐14) was used to assess cell viability. Antibodies directed against the CD45 (clone 30‐F11, Biolegend, 103149), CD3 (clone 17A2, BD, 740268), CD8 (clone 53−6.7, eBiosciences, 61‐0081‐82), and anti‐PD‐L1 (clone 10F.9G2, Biolegend, 124334) were added. Stained cells were analyzed with a Fortessa X20 cytometer (BD Biosciences).

### 4.17 Histology, immunohistochemistry, and immunofluorescence

Freshly collected tumour tissues were placed in 10% NBF and fixed for 24 h at RT followed by trimming to the thickness which did not exceed 3–5 mm. After rinsing with running water, the specimens were transferred to the vacuum tissue processor (HistoCore PEARL, Leica) for dehydration, then embedded into FFPE blocks using Tissue Embedding Center (EG1150, Leica). FFPE blocks were sectioned with a manual rotary microtome (RM2235, Leica), 4 µm thickness/section. Sections were processed for staining with hematoxylin and eosin (H&E), immunohistochemistry (IHC), or immunofluorescent (IF) analysis. For IHC, sections were stained with primary antibodies specific for anti‐PD‐L1 (ab174838F) and CD8 (#98941) or IF sections were stained with primary antibodies specific for CD31 (ab28364), NG2 (AB5320), and cell nuclei were counterstained with DAPI. All stained sections were scanned with Pannoramic Digital Slide Scanners for 40× magnification (3DHISTECH, Pannoramic SCAN). All the images were analyzed with HALO platform where tumour area and large areas of necrosis were quantified. Non‐tumour tissue on the periphery was excluded. Elongated blood vessels and pericyte co‐localization was quantified using Visiopharm platform. Quantitative histological analyses were performed at OracleBio Ltd. (Scotland, UK).

### 4.18 RT‐qPCR

Total RNA was extracted using the RNeasy Plus minikit (QIAGEN) following manufacturer's instructions and then reversed transcribed using iScript cDNA Synthesis Kit (Bio‐Rad). The primers used in this study are listed in Table 2 in the supplemental data. PCR program was used as following: 98°C for 30 s; then 40 cycles of 92°C for 1 s and 60°C for 15 s. Samples were run on LightCycler 480 system (Roche) using SYBR Green Master mix (QIAGEN). Reactions were performed with 25 ng of template cDNA. Transcript levels of genes were normalized to a reference index of housekeeping genes (18S ribosomal RNA and RPS2).

**TABLE 2:** RT‐qPCR primer sequences.

Gene	Forward sequence (5′−3′)	Reverse sequence (5′−3′)
VEGF	AGGGCAGAATCATCACGAAGT	AGGGTCTCGATTGGATGGCA
18srRNA	GATCAAAACCAACCCGGTCA	CCGTTTCTCAGGCTCCCTCT

### 4.19 Spheroid formation and treatment

HT‐29 spheroids were generated by seeding 1,500 HT‐29‐GFP cells with 1500 human colorectal cancer associated fibroblasts (CAF05) or human dermal fibroblasts (HDFs) or 3000 HT‐29GFP cells per well in ultra‐low attachment round bottom 96 well plates (Corning 7007). Spheroids were grown in tumour growth media, McCoy's media, supplemented with 10% FBS and 1% penicillin–streptomycin, with the presence of CD3/CD28 activated human PBMCs (effector‐to‐target cell ratios = 5:1) purchased from Cambridge Bioscience Ltd, and BioIVT, with stimulatory molecules anti‐CD3/anti‐CD28 (BD555336/555725, described previously). Treatments, including inactive compound control (1000 nm), and AD‐04 (1000 nm), were added to the co‐cultures from day 1. Size of spheroids was monitored using IncuCyte S3 imaging‐based system with Spheroid Module (Sartorius). Co‐cultures with treatments were maintained for 5−7 days for further analysis. siRNA knock‐down in spheroid co‐cultures: early passages of HDFs (P3–P4) were seeded in antibiotic‐free fibroblast growth media on day 1. SmartPool VEGFA siRNA (L‐003550‐00‐0010) and non‐targeting scrambled siRNA control (D‐001810‐01‐05) were purchased from Horizon Discovery Biosciences (Dharmacon); see Table 3. Transfection of siRNAs was performed on day 2 using Lipofectamine RNAiMAX (Invitrogen #13778‐150). 10 nm siRNA‐lipid complex was added onto cells and was incubated for 3 h at 37°. Fibroblast growth media was then added to the cells for a further incubation of 24 h. On day 3, siRNA transfected fibroblasts were activated by FGF2 and TGFβ for 48 h, followed by a VEGF ELISA assay to confirm knockdown efficiency. For spheroid co‐culture experiments, HDFs at 48 h post siRNA transfection were collected and co‐cultured with HT‐29‐GFP and activated primary human PBMCs, which were defrosted and rested for 18 h before co‐culture experiments started. Spheroid area (GFP fluorescent signal) was measured using the Sartorius Incucyte S3 spheroid module.

**TABLE 3:** siRNA sequences.

Target	siRNA number	sequence	Reference
hVEGF	1	GCAGAAUCAUCACGAAGUG	L‐003550‐00‐0010
hVEGF	2	CAACAAAUGUGAAUGCAGA	L‐003550‐00‐0010
hVEGF	3	GGAGUAXXXUGAUGAGAUC	L‐003550‐00‐0010
hVEGF	4	GAUCAAACCUCACCAAGGC	L‐003550‐00‐0010
Scrambled/ non‐targeting	1	UGGUUUACAUGUCGACUAA	D‐001810‐01‐05

### 4.20 RNAseq experiment and analysis

HDFs were treated with vehicle (DMSO) and AD‐04 for 6 and 24 h under hypoxic and normoxic conditions (group *n* = 5). Cells were harvested, frozen on dry ice and sent to the next generation sequencing CRO Genewiz for RNA‐extraction, RNA‐QC, and RNA‐sequencing (RNAseq). All treatment groups were compared to vehicle at the matching timepoint. RNAseq was performed on an Illumina Hi‐Seq machine using paired‐end 2×150 bp reads to a target coverage of approximately 40 million reads per sample. Data was resolved to .fastq file format and shipped to Almac Discovery for alignment and downstream analysis. Alignment was performed using the fast read aligner STAR^64^ producing .bam and .sam files against Ensembl *Homo sapiens* fasta genome build and genome definition file version GRCh38.87 was used to generate gene counts. Transcript‐specific read counts were resolved for each sample using StringTie.^65^ Normalization and statistical analysis of the gene‐level data was performed in R using the analysis package DESeq2^66^ and genome annotation package EnsDb.Hsapiens.v86 was used. Normalization and statistical analysis of the transcript‐level data was performed in R using the analysis package limma.^67^ Downstream canonical pathway, gene ontology enrichment and transcription factor enrichment analysis were performed in R using the enrichR^68^ toolkit. All bioinformatics data graphics were produced in R, producing high resolution .png images built using variations of the ggplot2 package functions.

## AUTHOR CONTRIBUTIONS

Anamarija Jurisic, Xavier Jacq, Aaron N. Cranston, Pei‐Ju Sung, Julien Daubriac, Gerald Gavory, Natalie Page, Daniel B. Longley and Timothy Harrison conceived the project. Aaron N. Cranston and Gerald Gavory designed the initial BioMAP experiments. Anamarija Jurisic, Pei‐Ju Sung, Julien Daubriac, Ian Lobb, Nyree Crawford and Eamon Cassidy performed the biology experiments. Mark Wappett and Wei‐Wei Kung performed the bioinformatics analyses. J.S. Shane Rountree, Matthew D. Helm, Timothy Harrison and Colin R. O'Dowd performed the medicinal chemistry that led to the invention of the USP7 inhibitors. Aaron N. Cranston, Pei‐Ju Sung, Anamarija Jurisic, Daniel B. Longley and Xavier Jacq designed and analyzed the in vivo experiments. Stephanie Feutren‐Burton and Aaron N. Cranston designed and analyzed the pharmacokinetic experiments. Richard D. Kennedy provided clinical positioning. Xavier Jacq and Anamarija Jurisic wrote the original manuscript draft. Pei‐Ju Sung, Aaron N. Cranston, Timothy Harrison, Daniel B. Longley, Nyree Crawford and Natalie Page reviewed and edited the manuscript. All authors approved the manuscript.

## ETHICS STATEMENT

All procedures and the use of materials and animals were performed according to appropriate, approved ethical guidelines.

## Supporting information

Supporting Information

## Data Availability

The data that support the findings of this study are available from the corresponding author upon reasonable request.

## References

[1] Hanahan D , Weinberg RA . Hallmarks of cancer: the next generation. Cell. 2011;144(5):646‐674. doi:10.1016/j.cell.2011.02.013 21376230

[2] Hanahan D , Coussens LM . Accessories to the crime: functions of cells recruited to the tumor microenvironment. Cancer Cell. 2012;21(3):309‐322. doi:10.1016/j.ccr.2012.02.022 22439926

[3] Junttila MR , de Sauvage FJ . Influence of tumour micro‐environment heterogeneity on therapeutic response. Nature. 2013;501(7467):346‐354. doi:10.1038/nature12626 24048067

[4] Restifo NP , Dudley ME , Rosenberg SA . Adoptive immunotherapy for cancer: harnessing the T cell response. Nat Rev Immunol. 2012;12(4):269‐281. doi:10.1038/nri3191 22437939 PMC6292222

[5] Prasad V , Kaestner V . Nivolumab and pembrolizumab: monoclonal antibodies against programmed cell death‐1 (PD‐1) that are interchangeable. Semin Oncol. 2017;44(2):132‐135. doi:10.1053/j.seminoncol.2017.06.007 28923211

[6] Sharma P , Hu‐Lieskovan S , Wargo JA , Primary RibasA . Adaptive, and acquired resistance to cancer immunotherapy. Cell. 2017;168(4):707‐723. doi:10.1016/j.cell.2017.01.017 28187290 PMC5391692

[7] Pugh CW , Ratcliffe PJ . Regulation of angiogenesis by hypoxia: role of the HIF system. Nat Med. 2003;9(6):677‐684. doi:10.1038/nm0603-677 12778166

[8] Kaelin WG Jr . The von Hippel‐Lindau protein, HIF hydroxylation, and oxygen sensing. Biochem Biophys Res Commun. 2005;338(1):627‐638. doi:10.1016/j.bbrc.2005.08.165 16153592

[9] Semenza GL . Targeting HIF‐1 for cancer therapy. Nat Rev Cancer. 2003;3(10):721‐732. doi:10.1038/nrc1187 13130303

[10] Wang GL , Jiang BH , Rue EA , Semenza GL . Hypoxia‐inducible factor 1 is a basic‐helix‐loop‐helix‐PAS heterodimer regulated by cellular O_2_ tension. Proc Natl Acad Sci U S A. 1995;92(12):5510‐5514. doi:10.1073/pnas.92.12.5510 7539918 PMC41725

[11] Salceda S , Caro J . Hypoxia‐inducible factor 1alpha (HIF‐1alpha) protein is rapidly degraded by the ubiquitin‐proteasome system under normoxic conditions. Its stabilization by hypoxia depends on redox‐induced changes. J Biol Chem. 1997;272(36):22642‐22647. doi:10.1074/jbc.272.36.22642 9278421

[12] Maxwell PH , Wiesener MS , Chang GW , et al. The tumour suppressor protein VHL targets hypoxia‐inducible factors for oxygen‐dependent proteolysis. Nature. 1999;399(6733):271‐275. doi:10.1038/20459 10353251

[13] Forsythe JA , Jiang BH , Iyer NV , et al. Activation of vascular endothelial growth factor gene transcription by hypoxia‐inducible factor 1. Mol Cell Biol. 1996;16(9):4604‐4613. doi:10.1128/mcb.16.9.4604 8756616 PMC231459

[14] Carmeliet P , Dor Y , Herbert JM , et al. Role of HIF‐1alpha in hypoxia‐mediated apoptosis, cell proliferation and tumour angiogenesis. Nature. 1998;394(6692):485‐490. doi:10.1038/28867 9697772

[15] Moreira IS , Fernandes PA , Ramos MJ . Vascular endothelial growth factor (VEGF) inhibition—a critical review. Anticancer Agents Med Chem. 2007;7(2):223‐245. doi:10.2174/187152007780058687 17348829

[16] Poon RT , Fan ST , Wong J . Clinical implications of circulating angiogenic factors in cancer patients. J Clin Oncol. 2001;19(4):1207‐1225. doi:10.1200/JCO.2001.19.4.1207 11181687

[17] Chen X , Song E . Turning foes to friends: targeting cancer‐associated fibroblasts. Nat Rev Drug Discovery. 2019;18(2):99‐115. doi:10.1038/s41573-018-0004-1 30470818

[18] Harrigan JA , Jacq X , Martin NM , Jackson SP . Deubiquitylating enzymes and drug discovery: emerging opportunities. Nat Rev Drug Discovery. 2018;17(1):57‐78. doi:10.1038/nrd.2017.152 28959952 PMC7097658

[19] Wertz IE , Murray JM . Structurally‐defined deubiquitinase inhibitors provide opportunities to investigate disease mechanisms. Drug Discovery Today Technol. 2019;31:109‐123. doi:10.1016/j.ddtec.2019.02.003 31200854

[20] Kategaya L , Di Lello P , Rouge L , et al. USP7 small‐molecule inhibitors interfere with ubiquitin binding. Nature. 2017;550(7677):534‐538. doi:10.1038/nature24006 29045385

[21] Turnbull AP , Ioannidis S , Krajewski WW , et al. Molecular basis of USP7 inhibition by selective small‐molecule inhibitors. Nature. 2017;550(7677):481‐486. doi:10.1038/nature24451 29045389 PMC6029662

[22] Lamberto I , Liu X , Seo HS , et al. Structure‐guided development of a potent and selective non‐covalent active‐site inhibitor of USP7. Cell Chem Biol. 2017;24(12):1490‐1500. doi:10.1016/j.chembiol.2017.09.003 29056421 PMC5749250

[23] O'Dowd CR , Helm MD , Rountree JSS , et al. Identification and structure‐guided development of pyrimidinone based USP7 inhibitors. ACS Med Chem Lett. 2018;9(3):238‐243. doi:10.1021/acsmedchemlett.7b00512 29541367 PMC5846043

[24] Gavory G , O'Dowd CR , Helm MD , et al. Discovery and characterization of highly potent and selective allosteric USP7 inhibitors. Nat Chem Biol. 2018;14(2):118‐125. doi:10.1038/nchembio.2528 29200206

[25] Tomasek JJ , Gabbiani G , Hinz B , Chaponnier C , Brown RA . Myofibroblasts and mechano‐regulation of connective tissue remodelling. Nat Rev Mol Cell Biol. 2002;3(5):349‐363. doi:10.1038/nrm809 11988769

[26] de Jong A , Merkx R , Berlin I , et al. Ubiquitin‐based probes prepared by total synthesis to profile the activity of deubiquitinating enzymes. Chembiochem. 2012;13(15):2251‐2258. doi:10.1002/cbic.201200497 23011887 PMC3487179

[27] Kallio PJ , Wilson WJ , O'Brien S , Makino Y , Poellinger L . Regulation of the hypoxia‐inducible transcription factor 1alpha by the ubiquitin‐proteasome pathway. J Biol Chem. 1999;274(10):6519‐6525. doi:10.1074/jbc.274.10.6519 10037745

[28] Mancini M , Magnani E , Macchi F , Bonapace IM . The multi‐functionality of UHRF1: epigenome maintenance and preservation of genome integrity. Nucleic Acids Res. 2021;49(11):6053‐6068. doi:10.1093/nar/gkab293 33939809 PMC8216287

[29] Achour M , Jacq X , Ronde P , et al. The interaction of the SRA domain of ICBP90 with a novel domain of DNMT1 is involved in the regulation of VEGF gene expression. Oncogene. 2008;27(15):2187‐2197. doi:10.1038/sj.onc.1210855 17934516

[30] Hurley JR , Balaji S , Narmoneva DA . Complex temporal regulation of capillary morphogenesis by fibroblasts. Am J Physiol Cell Physiol. 2010;299(2):C444‐C453. doi:10.1152/ajpcell.00572.2009 20505042 PMC3774339

[31] Kunz‐Schughart LA , Schroeder JA , Wondrak M , et al. Potential of fibroblasts to regulate the formation of three‐dimensional vessel‐like structures from endothelial cells in vitro. Am J Physiol Cell Physiol. 2006;290(5):C1385‐1398. doi:10.1152/ajpcell.00248.2005 16601149

[32] Berthod F , Germain L , Tremblay N , Auger FA . Extracellular matrix deposition by fibroblasts is necessary to promote capillary‐like tube formation in vitro. J Cell Physiol. 2006;207(2):491‐498. doi:10.1002/jcp.20584 16453301

[33] Motz GT , Coukos G . Deciphering and reversing tumor immune suppression. Immunity. 2013;39(1):61‐73. doi:10.1016/j.immuni.2013.07.005 23890064 PMC3782392

[34] Dirkx AE , Oude Egbrink MG , Kuijpers MJ , et al. Tumor angiogenesis modulates leukocyte‐vessel wall interactions in vivo by reducing endothelial adhesion molecule expression. Cancer Res. 2003;63(9):2322‐2329. 12727857

[35] Guerreiro N , Jullion A , Ferretti S , Fabre C , Meille C . Translational modeling of anticancer efficacy to predict clinical outcomes in a first‐in‐human phase 1 study of MDM2 inhibitor HDM201. AAPS J. 2021;23(2):28. doi:10.1208/s12248-020-00551-z 33554304

[36] Yang J , Yan J , Liu B . Targeting VEGF/VEGFR to modulate antitumor immunity. Front Immunol. 2018;9:978‐987. doi:10.3389/fimmu.2018.00978 29774034 PMC5943566

[37] Ohm JE , Carbone DP . VEGF as a mediator of tumor‐associated immunodeficiency. Immunol Res. 2001;23(2‐3):263‐272. doi:10.1385/IR:23:2-3:263 11444391

[38] Newman AC , Nakatsu MN , Chou W , Gershon PD , Hughes CCW . The requirement for fibroblasts in angiogenesis: Fibroblast‐derived matrix proteins are essential for endothelial cell lumen formation. Mol Biol Cell. 2011;22(20):3791‐3800. doi:10.1091/mbc.E11-05-0393 21865599 PMC3192859

[39] Ferrara N , Adamis AP . Ten years of anti‐vascular endothelial growth factor therapy. Nat Rev Drug Discovery. 2016;15(6):385‐403. doi:10.1038/nrd.2015.17 26775688

[40] Jain RK . Antiangiogenesis strategies revisited: from starving tumors to alleviating hypoxia. Cancer Cell. 2014;26(5):605‐622. doi:10.1016/j.ccell.2014.10.006 25517747 PMC4269830

[41] Vasudev NS , Reynolds AR . Anti‐angiogenic therapy for cancer: current progress, unresolved questions and future directions. Angiogenesis. 2014;17(3):471‐494. doi:10.1007/s10456-014-9420-y 24482243 PMC4061466

[42] Shojaei F , Wu X , Malik AK , et al. Tumor refractoriness to anti‐VEGF treatment is mediated by CD11b+Gr1+ myeloid cells. Nat Biotechnol. 2007;25(8):911‐920. doi:10.1038/nbt1323 17664940

[43] Rivera LB , Meyronet D , Hervieu V , Frederick MJ , Bergsland E , Bergers G . Intratumoral myeloid cells regulate responsiveness and resistance to antiangiogenic therapy. Cell Rep. 2015;11(4):577‐591. doi:10.1016/j.celrep.2015.03.055 25892230 PMC4438771

[44] Rivera LB , Bergers GCANCER . Tumor angiogenesis, from foe to friend. Science. 2015;349(6249):694‐695. doi:10.1126/science.aad0862 26273044

[45] Bouzin C , Brouet A , De Vriese J , Dewever J , Feron O . Effects of vascular endothelial growth factor on the lymphocyte‐endothelium interactions: identification of caveolin‐1 and nitric oxide as control points of endothelial cell anergy. J Immunol. 2007;178(3):1505‐1511. doi:10.4049/jimmunol.178.3.1505 17237399

[46] Manegold C , Dingemans AC , Gray JE , et al. The potential of combined immunotherapy and antiangiogenesis for the synergistic treatment of advanced NSCLC. J Thorac Oncol. 2017;12(2):194‐207. doi:10.1016/j.jtho.2016.10.003 27729297

[47] Bagaev A , Kotlov N , Nomie K , et al. Conserved pan‐cancer microenvironment subtypes predict response to immunotherapy. Cancer Cell. 2021;39(6):845‐865. doi:10.1016/j.ccell.2021.04.014 34019806

[48] van Loosdregt J , Fleskens V , Fu J , et al. Stabilization of the transcription factor Foxp3 by the deubiquitinase USP7 increases Treg‐cell‐suppressive capacity. Immunity. 2013;39(2):259‐271. doi:10.1016/j.immuni.2013.05.018 23973222 PMC4133784

[49] Wang F , Wang L , Wu J , et al. Active site‐targeted covalent irreversible inhibitors of USP7 impair the functions of Foxp3+ T‐regulatory cells by promoting ubiquitination of Tip60. PLoS One. 2017;12(12):e0189744. doi:10.1371/journal.pone.0189744 29236775 PMC5728538

[50] Wang L , Kumar S , Dahiya S , et al. Ubiquitin‐specific Protease‐7 inhibition impairs Tip60‐dependent Foxp3+ T‐regulatory cell function and promotes antitumor immunity. EBioMedicine. 2016;13:99‐112. doi:10.1016/j.ebiom.2016.10.018 27769803 PMC5264272

[51] Ritorto MS , Ewan R , Perez‐Oliva AB , et al. Screening of DUB activity and specificity by MALDI‐TOF mass spectrometry. Nat Commun. 2014;5:4763‐4774. doi:10.1038/ncomms5763 25159004 PMC4147353

[52] Bushman JW , Donovan KA , Schauer NJ , et al. Proteomics‐based identification of DUB substrates using selective inhibitors. Cell Chem Biol. 2020;28(1):78‐87. doi:10.1016/j.chembiol.2020.09.005 33007217 PMC7855594

[53] Wu HT , Kuo YC , Hung JJ , et al. K63‐polyubiquitinated HAUSP deubiquitinates HIF‐1alpha and dictates H3K56 acetylation promoting hypoxia‐induced tumour progression. Nat Commun. 2016;7:13644‐13661. doi:10.1038/ncomms13644 27934968 PMC5155157

[54] Lee JT , Gu W . The multiple levels of regulation by p53 ubiquitination. Cell Death Differ. 2010;17(1):86‐92. doi:10.1038/cdd.2009.77 19543236 PMC3690487

[55] Cummins JM , Rago C , Kohli M , Kinzler KW , Lengauer C , Vogelstein B . Tumour suppression: disruption of HAUSP gene stabilizes p53. Nature. 2004;428(6982):1‐2. doi:10.1038/nature02501 15058298

[56] An WG , Kanekal M , Simon MC , Maltepe E , Blagosklonny MV , Neckers LM . Stabilization of wild‐type p53 by hypoxia‐inducible factor 1alpha. Nature. 1998;392(6674):405‐408. doi:10.1038/32925 9537326

[57] Chen D , Li M , Luo J , Gu W . Direct interactions between HIF‐1 alpha and Mdm2 modulate p53 function. J Biol Chem. 2003;278(16):13595‐13598. doi:10.1074/jbc.C200694200 12606552

[58] Ravi R , Mookerjee B , Bhujwalla ZM , et al. Regulation of tumor angiogenesis by p53‐induced degradation of hypoxia‐inducible factor 1alpha. Genes Dev. 2000;14(1):34‐44. 10640274 PMC316350

[59] Nieminen AL , Qanungo S , Schneider EA , Jiang BH , Agani FH . Mdm2 and HIF‐1alpha interaction in tumor cells during hypoxia. J Cell Physiol. 2005;204(2):364‐369. doi:10.1002/jcp.20406 15880652

[60] Farhang Ghahremani M , Goossens S , Nittner D , et al. p53 promotes VEGF expression and angiogenesis in the absence of an intact p21‐Rb pathway. Cell Death Differ. 2013;20(7):888‐897. doi:10.1038/cdd.2013.12 23449391 PMC3679451

[61] Bronner C , Alhosin M , Hamiche A , Mousli M . Coordinated dialogue between UHRF1 and DNMT1 to ensure faithful inheritance of methylated DNA patterns. Genes (Basel). 2019;10(1):65‐79. doi:10.3390/genes10010065 30669400 PMC6360023

[62] Su D , Wang W , Hou Y , et al. Bimodal regulation of the PRC2 complex by USP7 underlies tumorigenesis. Nucleic Acids Res. 2021;49(8):4421‐4440. doi:10.1093/nar/gkab209 33849069 PMC8096222

[63] Felle M , Joppien S , Nemeth A , et al. The USP7/Dnmt1 complex stimulates the DNA methylation activity of Dnmt1 and regulates the stability of UHRF1. Nucleic Acids Res. 2011;39(19):8355‐8365. doi:10.1093/nar/gkr528 21745816 PMC3201865

[64] Dobin A , Davis CA , Schlesinger F , et al. STAR: ultrafast universal RNA‐seq aligner. Bioinformatics. 2013;29(1):15‐21. doi:10.1093/bioinformatics/bts635 23104886 PMC3530905

[65] Pertea M , Pertea GM , Antonescu CM , Chang TC , Mendell JT , Salzberg SL . StringTie enables improved reconstruction of a transcriptome from RNA‐seq reads. Nat Biotechnol. 2015;33(3):290‐295. doi:10.1038/nbt.3122 25690850 PMC4643835

[66] Love MI , Huber W , Anders S . Moderated estimation of fold change and dispersion for RNA‐seq data with DESeq2. Genome Biol. 2014;15(12):550‐571. doi:10.1186/s13059-014-0550-8 25516281 PMC4302049

[67] Ritchie ME , Phipson B , Wu D , et al. Limma powers differential expression analyses for RNA‐sequencing and microarray studies. Nucleic Acids Res. 2015;43(7):e47‐60. doi:10.1093/nar/gkv007 25605792 PMC4402510

[68] Kuleshov MV , Jones MR , Rouillard AD , et al. Enrichr: a comprehensive gene set enrichment analysis web server 2016 update. Nucleic Acids Res. 2016;44(W1):W90‐97. doi:10.1093/nar/gkw377 27141961 PMC4987924

