# Supplementary material for: USP7 inhibitors suppress tumour neoangiogenesis and promote synergy with immune checkpoint inhibitors by downregulating fibroblast VEGF
Source: Clin Transl Med. 2024 Apr 11;14(4):e1648. doi: 10.1002/ctm2.1648 (PMC11007818; doi:10.1002/ctm2.1648)
Supplement: Supplementary file 1 — Supporting Information [file CTM2-14-e1648-s001.docx]

**SUPPORTING INFORMATION**

**List of Supporting Information**

Chemical synthesis of ADC-159. (A) Reaction scheme and experimental protocols for the synthesis of ADC-159. (B) 500 MHz ^1^H NMR of ADC-159. **(C)** 126 MHz ^13^C NMR of ADC-159.

**Table S1 to S3**

Table S1. Selectivity of ADC-159 vs deubiquitinases at 10µM.

Table S2. Selectivity of ADC-159 vs kinases at 10µM.

Table S3. Selectivity of ADC-159 vs proteases at 10µM.

**Figure S1 to S8**

Fig. S1. Effect of USP7 inhibition on VEGF levels in activated fibroblasts.

Fig. S2. AD-04 USP7 target engagement in primary human fibroblasts and cancer cells.

Fig. S3. Modulation of sVEGF, proliferation, migration and invasion in response to USP7 and MDM2 inhibitors.

Fig. S4. Impact of USP7 inhibition on co-culture spheroid growth.

Movie S4.1. Spheroid formation of HT-29-GFP.

Movie S4.2. Spheroid formation of HT-29-GFP + fibroblasts.

Movie S4.3. Spheroid formation of HT-29-GFP + fibroblasts + PBMCs.

Fig. S5. Chemical structure, ADME properties and *in vitro* pharmacokinetic profile of ADC-159.

Fig. S6. Effect of USP7 on tumour microenvironment *in vivo.*

Fig. S7. Impact of USP7 inhibitor on anti-tumor immunity.

**Supporting Information: Chemical synthesis of ADC-159. (A)** Reaction scheme and experimental protocols for the synthesis of ADC-159. **(B)** 500 MHz ^1^H NMR of ADC-159. **(C)** 126 MHz ^13^C NMR of ADC-159.

**A**

*General experimental:* Organics solvents were purchased anhydrous from Sigma-Aldrich® in Sure/Seal™ bottles and were handled appropriately under nitrogen. Water was deionised using an Elga PURELAB Option-Q. All other solvents used (i.e. for work-up procedures and purification) were generally HPLC grade and were used as supplied from various commercial sources. All reagents were purchased from commercial suppliers and used as supplied. NMR spectra were recorded at ambient temperature using a Bruker Ascend (500 MHz) spectrometer. All chemical shifts (δ) are expressed in ppm. Residual solvent signals were used as an internal standard and the characteristic solvent peaks were corrected to the reference data outlined in *J. Org. Chem.*, 1997, 62, p7512-7515. Liquid Chromatography Mass Spectrometry (LCMS) experiments to determine retention times (R_T_) and associated mass ions were performed using the following methods:

Method A: The system consisted of an Agilent Technologies 6130 quadrupole mass spectrometer linked to an Agilent Technologies 1290 Infinity LC system with UV diode array detector and autosampler. The spectrometer consisted of an electrospray ionization source operating in positive and negative ion mode. LCMS experiments were performed on each sample submitted using the following conditions: LC Column: Agilent Eclipse Plus C18 RRHD, 1.8 µm, 50 x 2.1 mm maintained at 40 °C. Mobile phases: A) 0.1% (v/v) formic acid in water; B) 0.1% (v/v) formic acid in acetonitrile.

| Gradient Time (min) | Flow (mL/min) | %A | %B |
| --- | --- | --- | --- |
| 0.00 | 0.5 | 80 | 20 |
| 1.80 | 0.5 | 0 | 100 |
| 2.20 | 0.5 | 0 | 100 |
| 2.50 | 0.5 | 80 | 20 |
| 3.00 | 0.5 | 80 | 20 |

Method B: The system consisted of an Agilent Technologies 6140 single quadrupole mass spectrometer linked to an Agilent Technologies 1290 Infinity LC system with UV diode array detector and autosampler. The spectrometer consisted of a multimode ionization source (electrospray and atmospheric pressure chemical ionizations) operating in positive and negative ion mode. LCMS experiments were performed on each sample submitted using the following conditions: LC Column: Zorbax Eclipse Plus C18 RRHD, 1.8 µm, 50 x 2.1 mm maintained at 40 °C. Mobile phases: A) 0.1% (v/v) formic acid in water; B) 0.1% (v/v) formic acid in acetonitrile.

| Gradient Time (min) | Flow (mL/min) | %A | %B |
| --- | --- | --- | --- |
| 0.00 | 1.0 | 95 | 5 |
| 1.80 | 1.0 | 0 | 100 |
| 2.20 | 1.0 | 0 | 100 |
| 2.21 | 1.0 | 95 | 5 |
| 2.50 | 1.0 | 95 | 5 |

*Step 1:* A suspension of 4,6-dichloro-7*H*-pyrrolo[2,3-*d*]pyrimidine (500 mg, 2.66 mmol), (2,3-dihydrobenzofuran-5-yl)boronic acid (1.31 g, 7.98 mmol), 1,10-phenanthroline (958 mg, 5.32 mmol) and copper(II) acetate (966 mg, 5.32 mmol) in *N*,*N*-dimethylformamide (30 mL) was stirred at 20 °C under an atmosphere of air for 18 hours. The resulting mixture was diluted with ethyl acetate (100 mL) and washed with 1:1 brine/water (3 x 100 mL). The combined aqueous layers were back extracted with ethyl acetate (50 mL). The combined organic layers were dried (Phase Separator) and concentrated under reduced pressure. The resulting residue was purified by flash chromatography (0-40% ethyl acetate in cyclohexane) to give 4,6-dichloro-7-(2,3-dihydrobenzofuran-5-yl)-7*H*-pyrrolo[2,3-*d*]pyrimidine (392 mg, 46%) as a white solid. LCMS (Method A): R_T_ = 1.43 min, *m/z* = 306, 308 [M+H]^+^. ^1^H NMR (500 MHz, DMSO-*d*_6_) δ 8.61 (s, 1H, NC*H*N), 7.38 – 7.34 (m, 1H, CH_2_CC*H*CN), 7.21 (dd, J = 8.4, 2.3 Hz, 1H, CHC*H*CN), 7.04 (s, 1H, ClCC*H*), 6.95 (d, J = 8.4 Hz, 1H, OCC*H*CH), 4.65 (t, J = 8.7 Hz, 2H, CH_2_C*H_2_*O), 3.28 (t, J = 8.7 Hz, 2H, C*H*_2_CH_2_O).

*Step 2:* A suspension of 4,6-dichloro-7-(2,3-dihydrobenzofuran-5-yl)-7*H*-pyrrolo[2,3-*d*]pyrimidine (8.67 g, 28.3 mmol) in 1,4-dioxane (56.6 mL) and 2 M aqueous hydrochloric acid (56.6 mL, 113 mmol) was stirred at reflux under an atmosphere of nitrogen for 20 hours. Upon cooling to room temperature, the reaction mixture was diluted with water (80 mL) and the precipitate isolated by filtration. The precipitate was washed with water (3 x 80 mL) and dried in a vacuum oven at 50 °C to give 6-chloro-7-(2,3-dihydrobenzofuran-5-yl)-3,7-dihydro-4*H*-pyrrolo[2,3-*d*]pyrimidin-4-one (8.14 g, 99%) as a salmon pink solid. LCMS (Method B): R_T_ = 1.09 min, *m/z* = 288, 290 [M+H]^+^. ^1^H NMR (500 MHz, DMSO-*d*_6_) δ 12.12 (s, 1H, N*H*), 7.86 (s, 1H, NC*H*N), 7.29 – 7.24 (m, 1H, CH_2_CC*H*CN), 7.11 (dd, J = 8.4, 2.3 Hz, 1H, CHC*H*CN), 6.90 (d, J = 8.4 Hz, 1H, OCC*H*CH), 6.72 (s, 1H, ClCC*H*), 4.63 (t, J = 8.7 Hz, 2H, OC*H_2_*CH_2_), 3.26 (t, J = 8.7 Hz, 2H, OCH_2_C*H_2_*).

*Step 3:* A suspension of 6-chloro-7-(2,3-dihydrobenzofuran-5-yl)-3,7-dihydro-4*H*-pyrrolo[2,3-*d*]pyrimidin-4-one (357 mg, 1.04 mmol), *tert*-butyl 1-oxa-6-azaspiro[2.5]octane-6-carboxylate (442 mg, 2.08 mmol) and cesium carbonate (372 mg, 1.14 mmol) in *N*,*N*-dimethylformamide (8 mL) was stirred at heated at 80 °C for 4 hours. Upon cooling to room temperature, the reaction mixture was diluted with ethyl acetate (100 mL) and washed with brine (2 x 100 mL). The combined aqueous layers were back extracted with ethyl acetate (2 x 50 mL). The combined organic layers were dried (Phase Separator) and concentrated under reduced pressure. The resulting residue was purified by flash chromatography (0-80% ethyl acetate in cyclohexane) to give *tert*-butyl 4-((6-chloro-7-(2,3-dihydrobenzofuran-5-yl)-4-oxo-4,7-dihydro-3*H*-pyrrolo[2,3-*d*]pyrimidin-3-yl)methyl)-4-hydroxypiperidine-1-carboxylate (169 mg, 33%). LCMS (Method A): R_T_ = 1.45 min, *m/z* = 501 [M+H]^+^. ^1^H NMR (500 MHz, DMSO-*d*_6_) δ 8.06 (s, 1H, NC*H*N), 7.31 – 7.24 (m, 1H, CH_2_CC*H*CN), 7.12 (dd, J = 8.4, 2.3 Hz, 1H, CHC*H*CN), 6.91 (d, J = 8.4 Hz, 1H, OCC*H*CH), 6.75 (s, 1H, ClCC*H*), 4.86 (s, 1H, O*H*), 4.64 (t, J = 8.7 Hz, 2H), 3.99 (s, 2H), 3.73 – 3.52 (br. m, 2H), 3.26 (t, J = 8.7 Hz, 2H), 3.04 (br. s, 2H), 1.50 – 1.42 (m, 2H), 1.39 (s, 9H), 1.37 – 1.28 (m, 2H).

*Step 4:* A solution of *tert*-butyl 4-((6-chloro-7-(2,3-dihydrobenzofuran-5-yl)-4-oxo-4,7-dihydro-3*H*-pyrrolo[2,3-*d*]pyrimidin-3-yl)methyl)-4-hydroxypiperidine-1-carboxylate (169 mg, 0.337 mmol) in dichloromethane (3 mL) and trifluoroacetic acid (1.5 mL, 19.5 mmol) was stirred at room temperature for 1 hour. The reaction mixture was added to a 5 g pre-packed SCX-2 column pre-equilibrated with 20% methanol in dichloromethane. The bound product was washed with 20% methanol in dichloromethane (20 mL) and the resulting solution discarded. The bound product was eluted from the column using 20% 7N ammonia in methanol in dichloromethane (20 mL). The resulting solution containing the product was concentrated under reduced pressure to give 6-chloro-7-(2,3-dihydrobenzofuran-5-yl)-3-((4-hydroxypiperidin-4-yl)methyl)-3,7-dihydro-4*H*-pyrrolo[2,3-*d*]pyrimidin-4-one (118 mg, 86%) as an off-white solid. LCMS (Method A): R_T_ = 0.76 min, *m/z* = 401 [M+H]^+^.

*Step 5:* To a stirring solution of 6-chloro-7-(2,3-dihydrobenzofuran-5-yl)-3-((4-hydroxypiperidin-4-yl)methyl)-3,7-dihydro-4*H*-pyrrolo[2,3-*d*]pyrimidin-4-one (59.6 mg, 0.149 mmol), 1-methylcyclopropane-1-carboxylic acid (14.9 mg, 0.149 mmol) and *N*-[(Dimethylamino)-1*H*-1,2,3-triazolo-[4,5-*b*]pyridin-1-ylmethylene]-*N*-methylmethanaminium hexafluorophosphate *N*-oxide (67.8 mg, 0.178 mmol) in anhydrous dichloromethane (1 mL) at 20 °C was added *N*,*N*-diisopropylethylamine (78 µL, 0.446 mmol). The resulting solution was stirred for 30 min at 20 °C before the reaction mixture was washed with saturated aqueous sodium hydrogen carbonate (4 mL). The aqueous layer was extracted with dichloromethane (2 x 2 mL). The combined organic layers were dried (phase separator) and concentrated under reduced pressure. The resulting residue was purified by flash chromatography (50-100% EtOAc in Cyclohexane then 0-15% MeOH in EtOAc). The purified product was dissolved into a mixture of methanol (2 mL), acetonitrile (2 mL), and water (15 mL) before the resulting solution was freeze-dried to afford 6-chloro-7-(2,3-dihydrobenzofuran-5-yl)-3-((4-hydroxy-1-(1-methylcyclopropane-1-carbonyl)piperidin-4-yl)methyl)-3,7-dihydro-4*H*-pyrrolo[2,3-*d*]pyrimidin-4-one (**ADC-159**) (53.9 mg, 74%) as an amorphous white solid. LCMS (Method B): R_T_ = 1.08 min, *m/z* = 483 [M+H]^+^. ^1^H NMR (500 MHz, DMSO-*d*_6_) δ 8.07 (s, 1H, NC*H*N), 7.30 – 7.25 (m, 1H, CH_2_CC*H*CN), 7.15 – 7.09 (m, 1H, CHC*H*CN), 6.92 (d, *J* = 8.4 Hz, 1H, OCC*H*CH), 6.76 (s, 1H, ClC*H*C), 4.91 (s, 1H, O*H*), 4.64 (t, *J* = 8.7 Hz, 2H, OC*H_2_*CH_2_), 4.01 (s, 2H, NC*H_2_*COH), 3.95 (dt, *J* = 13.2, 4.1 Hz, 2H, CH_2_C*H_2_*N), 3.27 (t, *J* = 8.7 Hz, 2H, OCH_2_C*H_2_*), 3.16 (broad s, 2H, CH_2_C*H_2_*N), 1.57 – 1.45 (m, 2H, C*H_2_*CH_2_N), 1.45 – 1.34 (m, 2H, C*H_2_*CH_2_N), 1.21 (s, 3H, C*H_3_*), 0.81 – 0.70 (m, 2H, CH_3_CC*H_2_*), 0.57 – 0.46 (m, 2H, CH_3_CC*H_2_*). ^13^C NMR (126 MHz, DMSO) δ 171.24 (CH_2_N*C*OC), 160.10 (CH_2_CH_2_O*C*), 157.01 (CHC*C*ONCH), 148.32 (N*C*HN), 147.22 (ClCN*C*N), 128.50 (OCH_2_CH_2_*C*), 128.24 (CH*C*HCN), 126.13 (CH*C*NCCl), 125.34 (CH_2_C*C*HCN), 120.99 (Cl*CI*), 108.94 (OC*C*HCH), 106.60 (CClCH*C*CON), 100.79 (ClC*C*H), 71.74 (CH_2_*C*H_2_O), 69.25 (*C*OH), 53.20 (N*C*H_2_COH), 34.43 (NCH_2_*C*H_2_), 28.92 (OCH_2_*C*H_2_), 21.52 (*C*H_3_), 19.98 (CH_3_*C*), 12.16 (CH_3_C*C*H_2_). CH_2_*C*H_2_NCO not visible due to broad signal.

**B**

**C**

**Supporting Information Table S1: Selectivity of ADC-159 vs deubiquitinases at 10µM.**

| **Deubiquitinase** | **% Activity Remaining at 100µM** |
| --- | --- |
| USP1/UAF1 | 107 |
| USP2 | 81 |
| USP4 | 87 |
| USP5 | 74 |
| USP5 (+Ubiquitin@Kd) | 95 |
| USP5 (+Ubiquitin@Bmax) | 83 |
| USP6 | 90 |
| USP7 | -1 |
| USP8 | 75 |
| USP9x | 85 |
| USP11 | 89 |
| USP14 (Proteasome-VS@Kd) | 78 |
| USP15 | 93 |
| USP16 | 85 |
| USP19 | 70 |
| USP20 | 92 |
| USP21 | 80 |
| USP25 | 84 |
| USP27x | 77 |
| USP28 | 84 |
| USP30 | 87 |
| USP35 | 85 |
| USP36 | 86 |
| USP45 | 86 |
| CYLD | 80 |
| UCHL1 | 100 |
| UCHL3 | 96 |
| UCHL5 | 83 |
| BAP1 | 87 |
| OTU1 | 82 |
| OTUB2 | 89 |
| OTUD1 | 128 |
| OTUD3 | 94 |
| OTUD5 (p177S) | 75 |
| OTUD6A | 87 |
| OTUD6B | 96 |
| Cezanne | 85 |
| VCPIP | 75 |
| AMSH-LP | 83 |
| AMSH-LP (+Zinc) | 92 |
| Ataxin3 | 86 |
| Ataxin3L | 92 |
| JOSD1 | 83 |
| JOSD2 | 82 |

**Supporting Information Table S2: Selectivity of ADC-159 vs kinases at 10µM.**

| **Kinase** | **Assay Type** | **Km ATP** | **% Inhibition at 10µM** |
| --- | --- | --- | --- |
| AAK1 | Binding |  | -0.5 |
| ABL1 | Activity | Km app | -1.2 |
| ABL1 E255K | Activity | Km app | 0.2 |
| ABL1 F317I | Activity | Km app | -1.0 |
| ABL1 F317L | Activity | Km app | 2.0 |
| ABL1 G250E | Activity | Km app | 2.3 |
| ABL1 H396P | Binding |  | 12.6 |
| ABL1 M351T | Binding |  | 7.0 |
| ABL1 Q252H | Binding |  | 7.3 |
| ABL1 T315I | Activity | Km app | 2.6 |
| ABL1 Y253F | Activity | Km app | -5.8 |
| ABL2 (Arg) | Activity | Km app | 5.5 |
| ACVR1 (ALK2) | Binding |  | -0.4 |
| ACVR1 (ALK2) R206H | Binding |  | 0.5 |
| ACVR1B (ALK4) | Activity | Km app | 3.9 |
| ACVR2A | Binding |  | -7.9 |
| ACVR2B | Binding |  | 1.8 |
| ACVRL1 (ALK1) | Binding |  | 8.2 |
| ADCK3 | Binding |  | 6.6 |
| ADRBK1 (GRK2) | Activity | Km app | 1.8 |
| ADRBK2 (GRK3) | Activity | Km app | 3.3 |
| AKT1 (PKB alpha) | Activity | Km app | 4.4 |
| AKT2 (PKB beta) | Activity | Km app | -3.7 |
| AKT3 (PKB gamma) | Activity | Km app | 5.3 |
| ALK | Activity | Km app | 2.9 |
| ALK C1156Y | Binding |  | 18.3 |
| ALK F1174L | Binding |  | 1.9 |
| ALK L1196M | Binding |  | 5.5 |
| ALK R1275Q | Binding |  | 11.7 |
| ALK T1151_L1152insT | Binding |  | 3.4 |
| AMPK (A1/B1/G2) | Binding |  | 0.5 |
| AMPK (A1/B1/G3) | Binding |  | 4.5 |
| AMPK (A1/B2/G1) | Binding |  | -0.5 |
| AMPK (A1/B2/G2) | Activity | Km app | 2.4 |
| AMPK (A1/B2/G3) | Activity | Km app | 0.3 |
| AMPK (A2/B1/G2) | Activity | Km app | 7.5 |
| AMPK (A2/B1/G3) | Activity | Km app | 6.0 |
| AMPK (A2/B2/G1) | Binding |  | 2.5 |
| AMPK (A2/B2/G2) | Binding |  | -0.8 |
| AMPK (A2/B2/G3) | Activity | Km app | 3.2 |
| AMPK A1/B1/G1 | Activity | Km app | 6.8 |
| AMPK A2/B1/G1 | Activity | Km app | 4.2 |
| ANKK1 | Binding |  | 3.3 |
| AURKA (Aurora A) | Activity | Km app | 4.4 |
| AURKB (Aurora B) | Activity | Km app | 4.1 |
| AURKC (Aurora C) | Activity | Km app | 6.9 |
| AXL | Activity | Km app | 1.2 |
| AXL R499C | Binding |  | 2.4 |
| BLK | Activity | Km app | 3.9 |
| BMPR1A (ALK3) | Binding |  | 4.8 |
| BMPR1B (ALK6) | Binding |  | 1.1 |
| BMPR2 | Binding |  | 0.7 |
| BMX | Activity | Km app | 8.6 |
| BRAF | Binding |  | 7.8 |
| BRAF | Activity | 100 | 0.7 |
| BRAF V599E | Binding |  | 0.5 |
| BRAF V599E | Activity | 100 | 5.3 |
| BRSK1 (SAD1) | Activity | Km app | 5.5 |
| BRSK2 | Binding |  | 1.3 |
| BTK | Activity | Km app | 5.6 |
| CAMK1 (CaMK1) | Activity | 10 | 9.4 |
| CAMK1D (CaMKI delta) | Activity | Km app | 6.1 |
| CAMK1G (CAMKI gamma) | Activity | Km app | 0.6 |
| CAMK2A (CaMKII alpha) | Activity | Km app | 7.4 |
| CAMK2B (CaMKII beta) | Activity | Km app | 2.2 |
| CAMK2D (CaMKII delta) | Activity | Km app | 2.6 |
| CAMK2G (CaMKII gamma) | Binding |  | -3.0 |
| CAMK4 (CaMKIV) | Activity | Km app | 9.4 |
| CAMKK1 (CAMKKA) | Binding |  | 0.4 |
| CAMKK2 (CaMKK beta) | Binding |  | 2.8 |
| CASK | Binding |  | -2.7 |
| CDC42 BPA (MRCKA) | Activity | Km app | 5.4 |
| CDC42 BPB (MRCKB) | Activity | Km app | 3.6 |
| CDC42 BPG (MRCKG) | Activity | Km app | 3.0 |
| CDC7/DBF4 | Binding |  | 7.8 |
| CDK1/cyclin B | Activity | Km app | 4.2 |
| CDK11 (Inactive) | Binding |  | 0.8 |
| CDK11/cyclin C | Binding |  | 5.3 |
| CDK13/cyclin K | Binding |  | 0.9 |
| CDK14 (PFTK1)/cyclin Y | Binding |  | -10.8 |
| CDK16 (PCTK1)/cyclin Y | Binding |  | -2.9 |
| CDK17/cyclin Y | Activity | Km app | 1.9 |
| CDK18/cyclin Y | Activity | Km app | 6.7 |
| CDK2/cyclin A | Activity | Km app | 3.6 |
| CDK2/cyclin A1 | Binding |  | 3.9 |
| CDK2/cyclin E1 | Binding |  | -2.4 |
| CDK2/cyclin O | Binding |  | 3.7 |
| CDK3/cyclin E1 | Binding |  | 2.0 |
| CDK4/cyclin D1 | Activity | 10 | -2.2 |
| CDK4/cyclin D3 | Activity | 10 | 2.7 |
| CDK5 (Inactive) | Binding |  | -4.4 |
| CDK5/p25 | Activity | Km app | 1.7 |
| CDK5/p35 | Activity | Km app | 2.9 |
| CDK6/cyclin D1 | Activity | 10 | -1.4 |
| CDK7/cyclin H/MNAT1 | Activity | Km app | 0.0 |
| CDK8/cyclin C | Binding |  | -0.2 |
| CDK9 (Inactive) | Binding |  | 3.2 |
| CDK9/cyclin K | Binding |  | 0.9 |
| CDK9/cyclin T1 | Activity | Km app | 4.4 |
| CDKL5 | Activity | Km app | 1.9 |
| CHEK1 (CHK1) | Activity | Km app | -2.5 |
| CHEK2 (CHK2) | Activity | Km app | 4.0 |
| CHUK (IKK alpha) | Activity | Km app | -2.5 |
| CLK1 | Activity | Km app | 2.7 |
| CLK2 | Activity | Km app | 3.6 |
| CLK3 | Activity | Km app | 3.5 |
| CLK4 | Binding |  | 5.3 |
| CSF1R (FMS) | Activity | Km app | 10.7 |
| CSK | Activity | Km app | 2.3 |
| CSNK1A1 (CK1 alpha 1) | Activity | Km app | 13.3 |
| CSNK1A1L | Activity | Km app | 0.7 |
| CSNK1D (CK1 delta) | Activity | Km app | 6.7 |
| CSNK1E (CK1 epsilon) | Activity | Km app | 13.6 |
| CSNK1E (CK1 epsilon) R178C | Activity | Km app | 3.0 |
| CSNK1G1 (CK1 gamma 1) | Activity | Km app | 2.0 |
| CSNK1G2 (CK1 gamma 2) | Activity | Km app | 0.1 |
| CSNK1G3 (CK1 gamma 3) | Activity | Km app | 4.9 |
| CSNK2A1 (CK2 alpha 1) | Activity | Km app | 0.4 |
| CSNK2A2 (CK2 alpha 2) | Activity | Km app | 1.9 |
| DAPK1 | Activity | Km app | -3.8 |
| DAPK2 | Binding |  | 3.3 |
| DAPK3 (ZIPK) | Activity | Km app | 5.4 |
| DCAMKL1 (DCLK1) | Activity | Km app | 2.9 |
| DCAMKL2 (DCK2) | Activity | Km app | 2.5 |
| DDR1 | Binding |  | 0.2 |
| DDR2 | Binding |  | -1.7 |
| DDR2 N456S | Binding |  | -5.0 |
| DDR2 T654M | Binding |  | 1.5 |
| DMPK | Binding |  | -0.7 |
| DNA-PK | Activity | Km app | 6.4 |
| DYRK1A | Activity | Km app | 3.3 |
| DYRK1B | Activity | Km app | 8.9 |
| DYRK2 | Binding |  | 6.2 |
| DYRK3 | Activity | Km app | 5.5 |
| DYRK4 | Activity | Km app | 0.8 |
| EEF2K | Activity | Km app | 1.2 |
| EGFR (ErbB1) | Activity | Km app | 6.5 |
| EGFR (ErbB1) C797S | Activity | Km app | 3.2 |
| EGFR (ErbB1) G719C | Activity | Km app | 2.5 |
| EGFR (ErbB1) G719S | Activity | Km app | 2.7 |
| EGFR (ErbB1) L858R | Activity | Km app | 3.9 |
| EGFR (ErbB1) L861Q | Activity | Km app | 1.3 |
| EGFR (ErbB1) T790M | Activity | Km app | 3.8 |
| EGFR (ErbB1) T790M C797S L858R | Activity | Km app | 1.1 |
| EGFR (ErbB1) T790M L858R | Activity | Km app | 3.0 |
| EGFR (ErbB1) d746-750 | Binding |  | 2.5 |
| EGFR (ErbB1) d747-749 A750P | Binding |  | 14.7 |
| EIF2AK2 (PKR) | Binding |  | 5.7 |
| EPHA1 | Activity | Km app | 3.6 |
| EPHA2 | Activity | Km app | 6.9 |
| EPHA3 | Binding |  | 0.6 |
| EPHA4 | Activity | Km app | 7.3 |
| EPHA5 | Activity | Km app | 5.1 |
| EPHA6 | Binding |  | 1.2 |
| EPHA7 | Binding |  | 1.8 |
| EPHA8 | Activity | Km app | 4.4 |
| EPHB1 | Activity | Km app | 3.2 |
| EPHB2 | Activity | Km app | 7.1 |
| EPHB3 | Activity | Km app | 3.2 |
| EPHB4 | Activity | Km app | 7.1 |
| ERBB2 (HER2) | Activity | Km app | 1.6 |
| ERBB4 (HER4) | Activity | Km app | 5.2 |
| ERN1 | Binding |  | 2.1 |
| ERN2 | Binding |  | -1.5 |
| FER | Activity | Km app | 6.3 |
| FES (FPS) | Activity | Km app | 6.1 |
| FGFR1 | Activity | Km app | 2.8 |
| FGFR1 V561M | Binding |  | 3.1 |
| FGFR2 | Activity | Km app | -0.4 |
| FGFR2 N549H | Activity | Km app | 1.0 |
| FGFR3 | Activity | Km app | 5.7 |
| FGFR3 G697C | Binding |  | -3.3 |
| FGFR3 K650E | Activity | Km app | 3.7 |
| FGFR3 K650M | Binding |  | 1.4 |
| FGFR3 V555M | Activity | Km app | 3.5 |
| FGFR4 | Activity | Km app | -1.8 |
| FGR | Activity | Km app | 6.5 |
| FLT1 (VEGFR1) | Activity | Km app | -3.6 |
| FLT3 | Activity | Km app | 5.4 |
| FLT3 D835Y | Activity | Km app | 2.9 |
| FLT3 ITD | Binding |  | 3.9 |
| FLT4 (VEGFR3) | Activity | Km app | 1.1 |
| FRAP1 (mTOR) | Activity | Km app | 1.8 |
| FRK (PTK5) | Activity | Km app | 4.9 |
| FYN | Activity | Km app | 3.2 |
| FYN A | Binding |  | -0.7 |
| GAK | Binding |  | 2.4 |
| GRK1 | Binding |  | 0.4 |
| GRK4 | Activity | Km app | 5.0 |
| GRK5 | Activity | Km app | 0.0 |
| GRK6 | Activity | Km app | -0.8 |
| GRK7 | Activity | Km app | 0.1 |
| GSG2 (Haspin) | Activity | Km app | 0.4 |
| GSK3A (GSK3 alpha) | Activity | Km app | 4.2 |
| GSK3B (GSK3 beta) | Activity | Km app | 5.5 |
| HCK | Activity | Km app | 5.2 |
| HIPK1 (Myak) | Activity | Km app | 0.5 |
| HIPK2 | Activity | Km app | 0.9 |
| HIPK3 (YAK1) | Activity | Km app | 1.2 |
| HIPK4 | Activity | Km app | 4.1 |
| HUNK | Binding |  | -7.0 |
| ICK | Binding |  | 5.8 |
| IGF1R | Activity | Km app | 3.0 |
| IKBKB (IKK beta) | Activity | Km app | -1.3 |
| IKBKE (IKK epsilon) | Activity | Km app | -7.9 |
| INSR | Activity | Km app | 2.9 |
| INSRR (IRR) | Activity | Km app | 4.5 |
| IRAK1 | Activity | Km app | 0.1 |
| IRAK3 | Binding |  | 2.9 |
| IRAK4 | Activity | Km app | 0.6 |
| ITK | Activity | Km app | -2.3 |
| JAK1 | Activity | Km app | -0.1 |
| JAK2 | Activity | Km app | -1.4 |
| JAK2 JH1 JH2 | Activity | Km app | -0.1 |
| JAK2 JH1 JH2 V617F | Activity | Km app | 1.0 |
| JAK3 | Activity | Km app | -0.8 |
| KDR (VEGFR2) | Activity | Km app | 7.5 |
| KIT | Activity | Km app | 0.3 |
| KIT A829P | Binding |  | 6.1 |
| KIT D816H | Binding |  | 3.2 |
| KIT D816V | Binding |  | -0.7 |
| KIT D820E | Binding |  | 0.7 |
| KIT N822K | Binding |  | -0.4 |
| KIT T670E | Binding |  | -2.9 |
| KIT T670I | Activity | Km app | 1.3 |
| KIT V559D | Activity | Km app | -0.5 |
| KIT V559D T670I | Binding |  | 1.3 |
| KIT V559D V654A | Activity | Km app | 3.6 |
| KIT V560G | Activity | Km app | 0.7 |
| KIT V654A | Binding |  | 5.6 |
| KIT Y823D | Binding |  | 0.9 |
| KSR2 | Activity | Km app | 4.6 |
| LATS2 | Binding |  | 3.7 |
| LCK | Activity | Km app | 5.3 |
| LIMK1 | Binding |  | 1.7 |
| LIMK2 | Binding |  | 0.5 |
| LRRK2 | Activity | Km app | 5.4 |
| LRRK2 FL | Activity | Km app | 1.4 |
| LRRK2 G2019S | Activity | Km app | 1.9 |
| LRRK2 G2019S FL | Activity | Km app | 1.5 |
| LRRK2 I2020T | Activity | Km app | 4.1 |
| LRRK2 R1441C | Activity | Km app | 2.7 |
| LTK (TYK1) | Activity | Km app | 3.1 |
| LYN A | Activity | Km app | 3.0 |
| LYN B | Activity | Km app | 7.3 |
| MAP2K1 (MEK1) | Binding |  | -0.6 |
| MAP2K1 (MEK1) | Activity | 100 | 10.7 |
| MAP2K1 (MEK1) S218D S222D | Binding |  | -1.6 |
| MAP2K2 (MEK2) | Binding |  | 0.1 |
| MAP2K2 (MEK2) | Activity | 100 | 2.8 |
| MAP2K4 (MEK4) | Binding |  | -11.9 |
| MAP2K5 (MEK5) | Binding |  | 4.0 |
| MAP2K6 (MKK6) | Binding |  | -0.3 |
| MAP2K6 (MKK6) | Activity | 100 | 2.1 |
| MAP2K6 (MKK6) S207E T211E | Binding |  | 0.0 |
| MAP3K10 (MLK2) | Binding |  | 2.2 |
| MAP3K11 (MLK3) | Binding |  | -4.0 |
| MAP3K14 (NIK) | Binding |  | 1.5 |
| MAP3K19 (YSK4) | Activity | Km app | 7.4 |
| MAP3K2 (MEKK2) | Binding |  | 0.4 |
| MAP3K3 (MEKK3) | Binding |  | 0.1 |
| MAP3K5 (ASK1) | Binding |  | 10.9 |
| MAP3K7/MAP3K7IP1 (TAK1-TAB1) | Binding |  | -2.8 |
| MAP3K8 (COT) | Activity | 100 | 9.1 |
| MAP3K9 (MLK1) | Activity | Km app | -1.9 |
| MAP4K1 (HPK1) | Binding |  | 0.1 |
| MAP4K2 (GCK) | Activity | Km app | -5.1 |
| MAP4K3 (GLK) | Binding |  | -2.6 |
| MAP4K4 (HGK) | Activity | Km app | 2.7 |
| MAP4K5 (KHS1) | Activity | Km app | 4.6 |
| MAPK1 (ERK2) | Activity | Km app | 3.6 |
| MAPK10 (JNK3) | Binding |  | 3.5 |
| MAPK10 (JNK3) | Activity | 100 | 15.8 |
| MAPK11 (p38 beta) | Activity | Km app | 2.8 |
| MAPK12 (p38 gamma) | Activity | Km app | 11.2 |
| MAPK13 (p38 delta) | Activity | Km app | 2.4 |
| MAPK14 (p38 alpha) | Activity | 100 | 18.0 |
| MAPK14 (p38 alpha) Direct | Activity | Km app | 2.7 |
| MAPK15 (ERK7) | Binding |  | 0.4 |
| MAPK3 (ERK1) | Activity | Km app | 3.2 |
| MAPK7 (ERK5) | Activity | Km app | 4.2 |
| MAPK8 (JNK1) | Binding |  | 9.0 |
| MAPK8 (JNK1) | Activity | 100 | 15.4 |
| MAPK9 (JNK2) | Binding |  | 3.6 |
| MAPK9 (JNK2) | Activity | 100 | 9.8 |
| MAPKAPK2 | Activity | Km app | 5.3 |
| MAPKAPK3 | Activity | Km app | 4.5 |
| MAPKAPK5 (PRAK) | Activity | Km app | 3.8 |
| MARK1 (MARK) | Activity | Km app | 3.6 |
| MARK2 | Activity | Km app | 8.2 |
| MARK3 | Activity | Km app | 5.9 |
| MARK4 | Activity | Km app | 0.8 |
| MASTL | Binding |  | 2.9 |
| MATK (HYL) | Activity | Km app | 5.8 |
| MELK | Activity | Km app | 12.8 |
| MERTK (cMER) | Activity | Km app | 3.8 |
| MERTK (cMER) A708S | Binding |  | -1.5 |
| MET (cMet) | Activity | Km app | -0.7 |
| MET (cMet) Y1235D | Activity | Km app | 4.0 |
| MET D1228H | Binding |  | -4.6 |
| MET M1250T | Activity | Km app | -0.1 |
| MINK1 | Activity | Km app | 7.3 |
| MKNK1 (MNK1) | Activity | Km app | 2.4 |
| MKNK2 (MNK2) | Binding |  | 0.3 |
| MLCK (MLCK2) | Binding |  | -2.5 |
| MLK4 | Binding |  | 1.4 |
| MST1R (RON) | Activity | Km app | -0.4 |
| MST4 | Activity | Km app | 7.6 |
| MUSK | Activity | Km app | 3.8 |
| MYLK (MLCK) | Binding |  | -0.1 |
| MYLK2 (skMLCK) | Activity | Km app | 4.8 |
| MYLK4 | Binding |  | 8.7 |
| MYO3A (MYO3 alpha) | Binding |  | -10.0 |
| MYO3B (MYO3 beta) | Binding |  | -5.7 |
| NEK1 | Activity | Km app | 9.4 |
| NEK2 | Activity | Km app | -5.2 |
| NEK4 | Activity | Km app | 6.8 |
| NEK6 | Activity | Km app | 1.8 |
| NEK8 | Binding |  | -4.9 |
| NEK9 | Activity | Km app | 4.2 |
| NIM1K | Activity | Km app | 2.9 |
| NLK | Binding |  | 4.2 |
| NTRK1 (TRKA) | Activity | Km app | 9.9 |
| NTRK2 (TRKB) | Activity | Km app | 5.5 |
| NTRK3 (TRKC) | Activity | Km app | 3.2 |
| NUAK1 (ARK5) | Activity | Km app | 8.6 |
| NUAK2 | Binding |  | 1.8 |
| PAK1 | Activity | Km app | 3.9 |
| PAK2 (PAK65) | Activity | Km app | 1.6 |
| PAK3 | Activity | Km app | 8.3 |
| PAK4 | Activity | Km app | 1.4 |
| PAK6 | Activity | Km app | 2.4 |
| PAK7 (KIAA1264) | Activity | Km app | 5.3 |
| PASK | Activity | Km app | 2.2 |
| PDGFRA (PDGFR alpha) | Activity | Km app | -1.9 |
| PDGFRA D842V | Activity | Km app | 1.0 |
| PDGFRA T674I | Activity | Km app | 4.4 |
| PDGFRA V561D | Activity | Km app | 1.3 |
| PDGFRB (PDGFR beta) | Activity | Km app | 1.4 |
| PDK1 | Activity | 100 | 7.2 |
| PDK1 Direct | Activity | Km app | 1.0 |
| PEAK1 | Activity | Km app | 3.2 |
| PHKG1 | Activity | Km app | -0.3 |
| PHKG2 | Activity | Km app | -3.8 |
| PI4K2A (PI4K2 alpha) | Activity | Km app | 1.0 |
| PI4K2B (PI4K2 beta) | Activity | Km app | 2.5 |
| PI4KA (PI4K alpha) | Activity | 10 | -0.9 |
| PI4KB (PI4K beta) | Activity | Km app | 5.4 |
| PIK3C2A (PI3K-C2 alpha) | Activity | Km app | 2.2 |
| PIK3C2B (PI3K-C2 beta) | Activity | 10 | -2.6 |
| PIK3C2G (PI3K-C2 gamma) | Activity | Km app | 6.7 |
| PIK3C3 (hVPS34) | Activity | Km app | 1.9 |
| PIK3CA E542K/PIK3R1 (p110 alpha E542K/p85 alpha) | Activity | 10 | -6.0 |
| PIK3CA E545K/PIK3R1 (p110 alpha E545K/p85 alpha) | Activity | Km app | 1.4 |
| PIK3CA/PIK3R1 (p110 alpha/p85 alpha) | Activity | Km app | -0.4 |
| PIK3CA/PIK3R3 (p110 alpha/p55 gamma) | Activity | Km app | -1.3 |
| PIK3CB/PIK3R1 (p110 beta/p85 alpha) | Activity | Km app | -18.2 |
| PIK3CB/PIK3R2 (p110 beta/p85 beta) | Activity | Km app | -2.3 |
| PIK3CD/PIK3R1 (p110 delta/p85 alpha) | Activity | Km app | 5.0 |
| PIK3CG (p110 gamma) | Activity | Km app | 2.8 |
| PIM1 | Activity | Km app | -0.1 |
| PIM2 | Activity | Km app | 0.0 |
| PIM3 | Activity | Km app | -5.9 |
| PIP4K2A | Activity | 10 | -13.8 |
| PIP5K1A | Activity | 10 | -1.3 |
| PIP5K1B | Activity | 10 | -1.1 |
| PIP5K1C | Activity | 10 | -2.1 |
| PKMYT1 | Binding |  | -1.2 |
| PKN1 (PRK1) | Activity | Km app | 0.0 |
| PKN2 (PRK2) | Binding |  | 1.7 |
| PLK1 | Activity | Km app | 1.7 |
| PLK2 | Activity | Km app | 2.9 |
| PLK3 | Activity | Km app | -6.2 |
| PLK4 | Binding |  | 0.8 |
| PRKACA (PKA) | Activity | Km app | 1.0 |
| PRKACB (PRKAC beta) | Binding |  | -1.3 |
| PRKACG (PRKAC gamma) | Binding |  | 1.9 |
| PRKCA (PKC alpha) | Activity | Km app | 5.6 |
| PRKCB1 (PKC beta I) | Activity | Km app | 25.5 |
| PRKCB2 (PKC beta II) | Activity | Km app | 7.9 |
| PRKCD (PKC delta) | Activity | Km app | 5.8 |
| PRKCE (PKC epsilon) | Activity | Km app | 5.5 |
| PRKCG (PKC gamma) | Activity | Km app | 8.0 |
| PRKCH (PKC eta) | Activity | Km app | 0.9 |
| PRKCI (PKC iota) | Activity | Km app | 1.7 |
| PRKCN (PKD3) | Activity | Km app | 4.7 |
| PRKCQ (PKC theta) | Activity | Km app | -2.8 |
| PRKCZ (PKC zeta) | Activity | Km app | -0.2 |
| PRKD1 (PKC mu) | Activity | Km app | 5.4 |
| PRKD2 (PKD2) | Activity | Km app | 3.2 |
| PRKG1 | Activity | Km app | 3.7 |
| PRKG2 (PKG2) | Activity | Km app | 1.1 |
| PRKX | Activity | Km app | 5.6 |
| PTK2 (FAK) | Activity | Km app | 3.3 |
| PTK2B (FAK2) | Activity | Km app | 5.5 |
| PTK6 (Brk) | Activity | Km app | 1.1 |
| RAF1 (cRAF) Y340D Y341D | Binding |  | 0.7 |
| RAF1 (cRAF) Y340D Y341D | Activity | 100 | 5.8 |
| RET | Activity | Km app | 0.4 |
| RET A883F | Activity | Km app | -0.3 |
| RET G691S | Binding |  | 0.2 |
| RET M918T | Binding |  | -2.3 |
| RET S891A | Activity | Km app | 0.5 |
| RET V804E | Activity | Km app | -0.1 |
| RET V804L | Activity | Km app | 3.4 |
| RET V804M | Binding |  | 0.8 |
| RET Y791F | Activity | Km app | 3.4 |
| RIPK2 | Binding |  | 1.0 |
| RIPK3 | Binding |  | 9.5 |
| ROCK1 | Activity | Km app | -4.2 |
| ROCK2 | Activity | Km app | 3.2 |
| ROS1 | Activity | Km app | 3.8 |
| RPS6KA1 (RSK1) | Activity | Km app | 3.7 |
| RPS6KA2 (RSK3) | Activity | Km app | 10.3 |
| RPS6KA3 (RSK2) | Activity | Km app | 4.6 |
| RPS6KA4 (MSK2) | Activity | Km app | 4.1 |
| RPS6KA5 (MSK1) | Activity | Km app | 1.6 |
| RPS6KA6 (RSK4) | Activity | Km app | 5.4 |
| RPS6KB1 (p70S6K) | Activity | Km app | 1.3 |
| RPS6KB2 (p70S6Kb) | Activity | Km app | 0.1 |
| SBK1 | Activity | Km app | 2.8 |
| SGK (SGK1) | Activity | Km app | 6.1 |
| SGK2 | Activity | Km app | 2.4 |
| SGKL (SGK3) | Activity | Km app | -2.6 |
| SIK1 | Binding |  | 13.4 |
| SIK3 | Binding |  | 2.6 |
| SLK | Binding |  | -0.3 |
| SNF1LK2 | Activity | Km app | 4.6 |
| SPHK1 | Activity | Km app | -3.7 |
| SPHK2 | Activity | 10 | 2.8 |
| SRC | Activity | Km app | 3.8 |
| SRC N1 | Activity | Km app | 3.8 |
| SRMS (Srm) | Activity | Km app | 3.2 |
| SRPK1 | Activity | Km app | 3.0 |
| SRPK2 | Activity | Km app | -1.3 |
| STK16 (PKL12) | Binding |  | 1.6 |
| STK17A (DRAK1) | Binding |  | 5.3 |
| STK17B (DRAK2) | Binding |  | 4.6 |
| STK22B (TSSK2) | Activity | Km app | 2.9 |
| STK22D (TSSK1) | Activity | Km app | 2.3 |
| STK23 (MSSK1) | Activity | Km app | 3.9 |
| STK24 (MST3) | Activity | Km app | 7.0 |
| STK25 (YSK1) | Activity | Km app | -0.1 |
| STK3 (MST2) | Activity | Km app | 4.0 |
| STK32B (YANK2) | Binding |  | -0.3 |
| STK32C (YANK3) | Binding |  | 3.4 |
| STK33 | Binding |  | -0.3 |
| STK38 (NDR) | Binding |  | -2.2 |
| STK38L (NDR2) | Binding |  | -0.6 |
| STK39 (STLK3) | Binding |  | -0.8 |
| STK4 (MST1) | Activity | Km app | -0.3 |
| SYK | Activity | Km app | -0.9 |
| TAOK1 | Binding |  | -1.1 |
| TAOK2 (TAO1) | Activity | Km app | 0.1 |
| TAOK3 (JIK) | Binding |  | 3.3 |
| TBK1 | Activity | Km app | 2.3 |
| TEC | Binding |  | 1.4 |
| TEK (TIE2) R849W | Binding |  | -1.1 |
| TEK (TIE2) Y1108F | Binding |  | 3.9 |
| TEK (TIE2) Y897S | Activity | Km app | 5.5 |
| TEK (Tie2) | Activity | Km app | 15.7 |
| TESK1 | Binding |  | -1.8 |
| TESK2 | Binding |  | 1.8 |
| TGFBR1 (ALK5) | Binding |  | 0.2 |
| TGFBR2 | Binding |  | 6.8 |
| TLK1 | Binding |  | -2.1 |
| TLK2 | Binding |  | 0.1 |
| TNIK | Binding |  | 6.8 |
| TNK1 | Activity | Km app | 0.0 |
| TNK2 (ACK) | Binding |  | 2.4 |
| TTK | Binding |  | 6.1 |
| TXK | Activity | Km app | 1.2 |
| TYK2 | Activity | Km app | 5.5 |
| TYRO3 (RSE) | Activity | Km app | 5.2 |
| ULK1 | Binding |  | -6.5 |
| ULK2 | Binding |  | -0.2 |
| ULK3 | Binding |  | 6.6 |
| VRK2 | Binding |  | 4.3 |
| WEE1 | Binding |  | -2.0 |
| WNK1 | Binding |  | -0.7 |
| WNK2 | Binding |  | -0.3 |
| WNK3 | Binding |  | -2.6 |
| YES1 | Activity | Km app | 6.5 |
| ZAK | Binding |  | 1.4 |
| ZAP70 | Activity | Km app | 5.4 |

**Supporting Information Table S3: Selectivity of ADC-159 vs proteases at 10µM.**

| **Protease** | **% Enzyme Activity (relative to DMSO controls)** | | **Control Compound IC_50_ (M)** | **Control compound ID** |
| --- | --- | --- | --- | --- |
|  | **ADC-159 (10µM)** | |  |  |
|  | **Data 1** | **Data 2** |  |  |
| ACE1 | 106.5 | 106.8 | 4.7E-08 | Captopril |
| ACE2 | 97.5 | 96.1 | 6.54E-10 | ACE 2 inhibitor |
| ADAM10 | 108.4 | 108.7 | 1.4E-06 | GM6001 |
| BACE1 | 102.2 | 105.3 | 1.71E-07 | B-Secretase inhibitor IV |
| Calpain 1 | 100.4 | 99.5 | 1.4E-08 | E64 |
| Caspase 1 | 87.1 | 88.3 | 1.93E-08 | IETD-CHO |
| Caspase 2 | 73.4 | 73.7 | 2.57E-07 | IETD-CHO |
| Caspase 3 | 103.1 | 102.9 | 7.58E-10 | DEVD-CHO |
| Caspase 4 | 74.0 | 74.2 | 2.61E-06 | IETD-CHO |
| Caspase 5 | 76.0 | 76.5 | 7.45E-09 | IETD-CHO |
| Caspase 6 | 102.0 | 100.1 | 3.63E-08 | DEVD-CHO |
| Caspase 7 | 99.7 | 99.0 | 1.58E-09 | DEVD-CHO |
| Caspase 8 | 84.1 | 83.3 | 3.97E-09 | IETD-CHO |
| Caspase 9 | 29.7 | 30.2 | 1.37E-08 | IETD-CHO |
| Caspase 10 | 133.6 | 134.3 | 1.04E-08 | IETD-CHO |
| Caspase 11 | 74.8 | 74.2 | 3.18E-07 | IETD-CHO |
| Caspase 14 | 88.3 | 88.6 | 3.94E-08 | WEHD-CHO |
| Cathepsin B | 109.7 | 109.9 | 4.14E-09 | E64 |
| Cathepsin C | 109.6 | 106.8 | 1.22E-06 | E64 |
| Cathepsin D | 90.8 | 90.9 | 1.66E-10 | Pepstatin A |
| Cathepsin E | 102.5 | 102.2 | 1.28E-10 | Pepstatin A |
| Cathepsin G | 95.1 | 94.1 | 3.44E-06 | Chymostatin |
| Cathepsin H | 101.0 | 100.7 | 1.98E-08 | E64 |
| Cathepsin K | 95.8 | 94.6 | 7.52E-10 | E64 |
| Cathepsin L | 101.8 | 101.8 | 1.88E-09 | E64 |
| Cathepsin S | 79.3 | 80.4 | 8.97E-10 | E64 |
| Cathepsin V | 104.3 | 104.7 | 4.35E-09 | E64 |
| Chymase | 95.4 | 96.8 | 1.02E-08 | Chymostatin |
| Chymotrypsin | 99.6 | 98.3 | 4.01E-10 | Chymostatin |
| DPP IV | 104.1 | 105.5 | 3.02E-07 | P32/98 |
| DPP VIII | 87.4 | 88.7 | 2.08E-06 | P32/98 |
| DPP IX | 96.1 | 96.0 | 1.71E-06 | P32/98 |
| Elastase | 102.3 | 101.0 | 6.73E-09 | Sivelestat |
| Furin | 102.4 | 104.9 | 1.41E-09 | Furin Inhibitor I |
| FVIIa | 102.1 | 104.1 | 1.21E-07 | PCI 27483 |
| FXa | 104.4 | 105.0 | 2.88E-06 | Gabexate mesylate (GM) |
| FXIa | 104.5 | 104.2 | 4.37E-07 | Gabexate mesylate (GM) |
| HIV-1 | 108.9 | 108.3 | 7.81E-06 | Pepstatin A |
| Kallikrein 1 | 100.5 | 99.3 | 4.14E-06 | Leupeptin |
| Kallikrein 5 | 102.3 | 104.6 | 1.13E-05 | Gabexate mesylate (GM) |
| Kallikrein 7 | 90.9 | 90.1 | 2.62E-05 | Gabexate mesylate (GM) |
| Kallikrein 12 | 99.3 | 99.1 | 1.19E-07 | Gabexate mesylate (GM) |
| Kallikrein 13 | 95.5 | 96.4 | 1.68E-05 | Gabexate mesylate (GM) |
| Kallikrein 14 | 116.5 | 115.5 | 6.86E-07 | Gabexate mesylate (GM) |
| Matriptase 2 | 101.8 | 99.2 | 1.11E-06 | Gabexate mesylate (GM) |
| MMP 1 | 96.3 | 96.0 | 2.31E-10 | GM6001 |
| MMP 2 | 89.8 | 90.7 | 1.41E-10 | GM6001 |
| MMP 3 | 96.2 | 96.0 | 3.18E-08 | GM6001 |
| MMP 7 | 94.1 | 94.7 | 1.19E-08 | GM6001 |
| MMP 8 | 99.7 | 99.7 | 3.22E-10 | GM6001 |
| MMP 9 | 94.5 | 95.4 | 1.64E-10 | GM6001 |
| MMP 10 | 98.0 | 97.6 | 2.19E-08 | GM6001 |
| MMP 12 | 86.7 | 86.5 | 2.34E-10 | GM6001 |
| MMP 13 | 96.1 | 95.6 | 7.71E-11 | GM6001 |
| MMP 14 | 89.8 | 90.0 | 7.62E-10 | GM6001 |
| Neprilysin | 103.3 | 103.1 | 1.5E-07 | Phosphoramidon |
| Papain | 98.1 | 97.9 | 1.16E-10 | E64 |
| Plasma Kallikrein | 107.7 | 104.7 | 2.42E-07 | Gabexate mesylate (GM) |
| Plasmin | 105.3 | 106.3 | 4.93E-07 | Gabexate mesylate (GM) |
| Proteinase A | 105.4 | 106.5 | 0.000113 | Leupeptin |
| Proteinase K | 100.2 | 100.6 | 2.46E-07 | Proteinase K inhibitor |
| SARS-CoV2-Mpro | 101.5 | 101.9 | 2.36E-08 | GC376 |
| SARS-CoV2-Plpro | 102.6 | 103.5 | 1.02E-06 | Plpro inhibitor |
| TACE | 104.4 | 105.2 | 4.06E-08 | GM6001 |
| Thrombin a | 103.3 | 103.5 | 8.31E-07 | Gabexate mesylate (GM) |
| TMPRSS2 | 101.0 | 101.1 | 1.39E-09 | Camostat |
| Trypsin | 103.5 | 103.3 | 1.91E-08 | Gabexate mesylate (GM) |
| Tryptase b2 | 90.5 | 88.0 | 8.01E-09 | Gabexate mesylate (GM) |
| Tryptase g1 | 102.2 | 102.1 | 1.04E-08 | Gabexate mesylate (GM) |
| Urokinase | 96.7 | 97.4 | 3.68E-08 | Gabexate mesylate (GM) |

**Supporting Information Figure S1.**

**
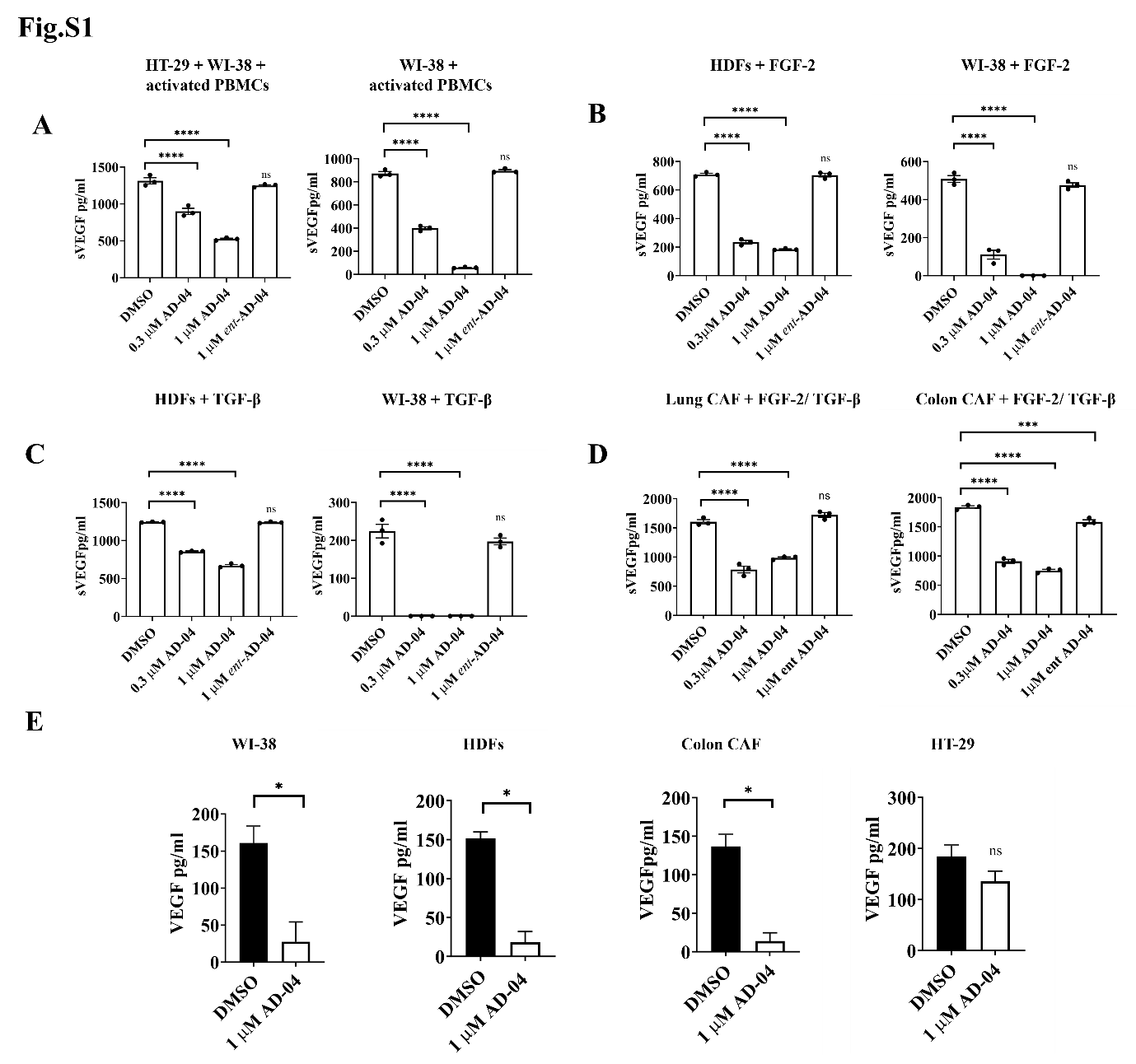
**

**Figure S1. Effect of USP7 inhibition on VEGF levels in activated fibroblasts.** Impact of AD-04 on VEGF secretion from (A) HT-29 cells co-cultured with WI-38 fibroblasts and activated PBMCs (left), and WI-38 fibroblasts and activated PBMCs (right). (B) Fibroblasts, HDFs (left) and WI-38 (right), activated with FGF-2. (C) Fibroblasts, HDFs (left) and WI-38 (right) activated with TGF-β. (D) FGF-2 activated primary fibroblasts (WI-38 and HDFs), colon CAFs cancer cells (HT-29) were treated with AD-04 and *ent*-AD-04 for 48h and intracellular VEGF measured by ELISA. Data presented as mean +/- S.E.M., * p=0.01 (unpaired t-test), ns not significant.

**Supporting Information Figure S2.**

**
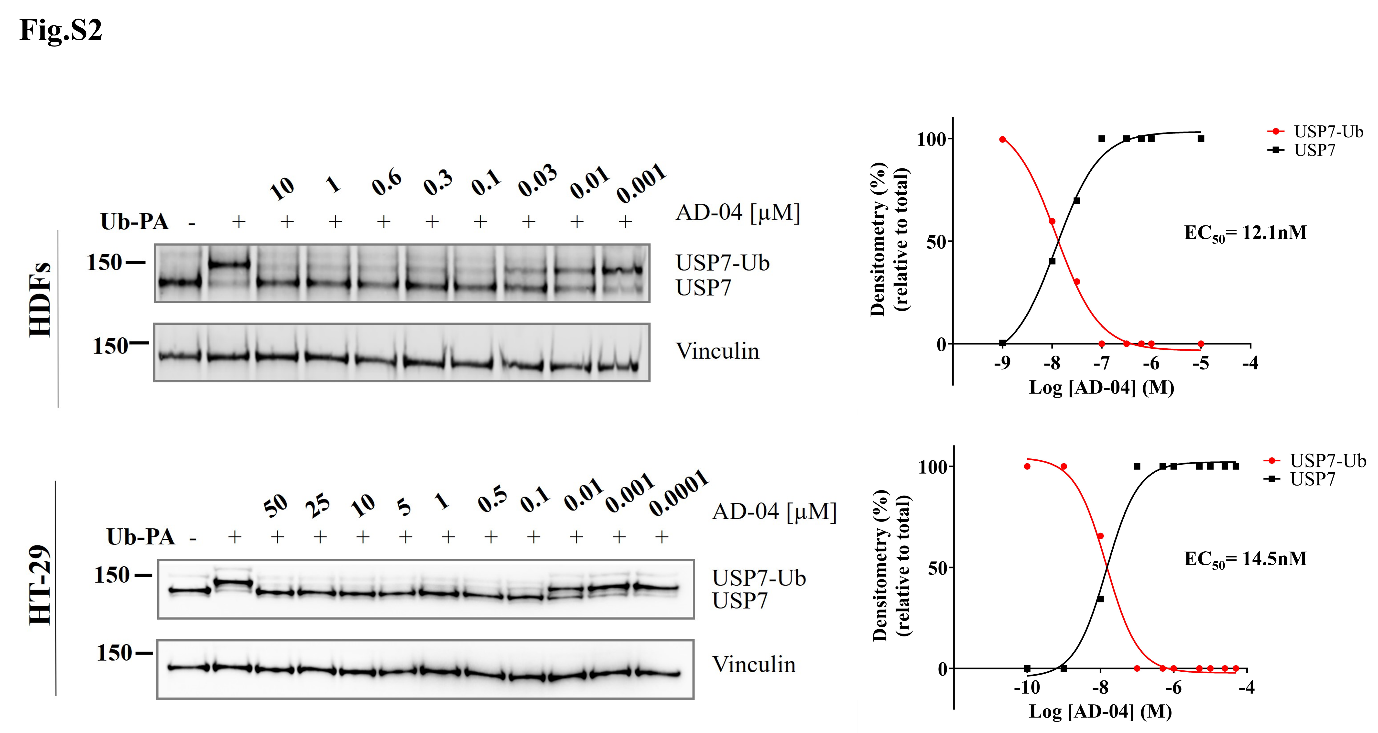
**

**Figure S2. AD-04 USP7 target engagement in primary human fibroblasts and cancer cells.** Lysates from HDF cells (top) or HT-29 cancer cells (bottom) were treated for 2h with AD-04 or *ent*-AD-04 were incubated with ubiquitin-proparglylamine (Ub-PA) activity probe. Samples were analyzed by immuno-blotting using USP7 antibody. EC_50_ values were determined by densitometry analysis.

**Supporting Information Figure S3.**

**
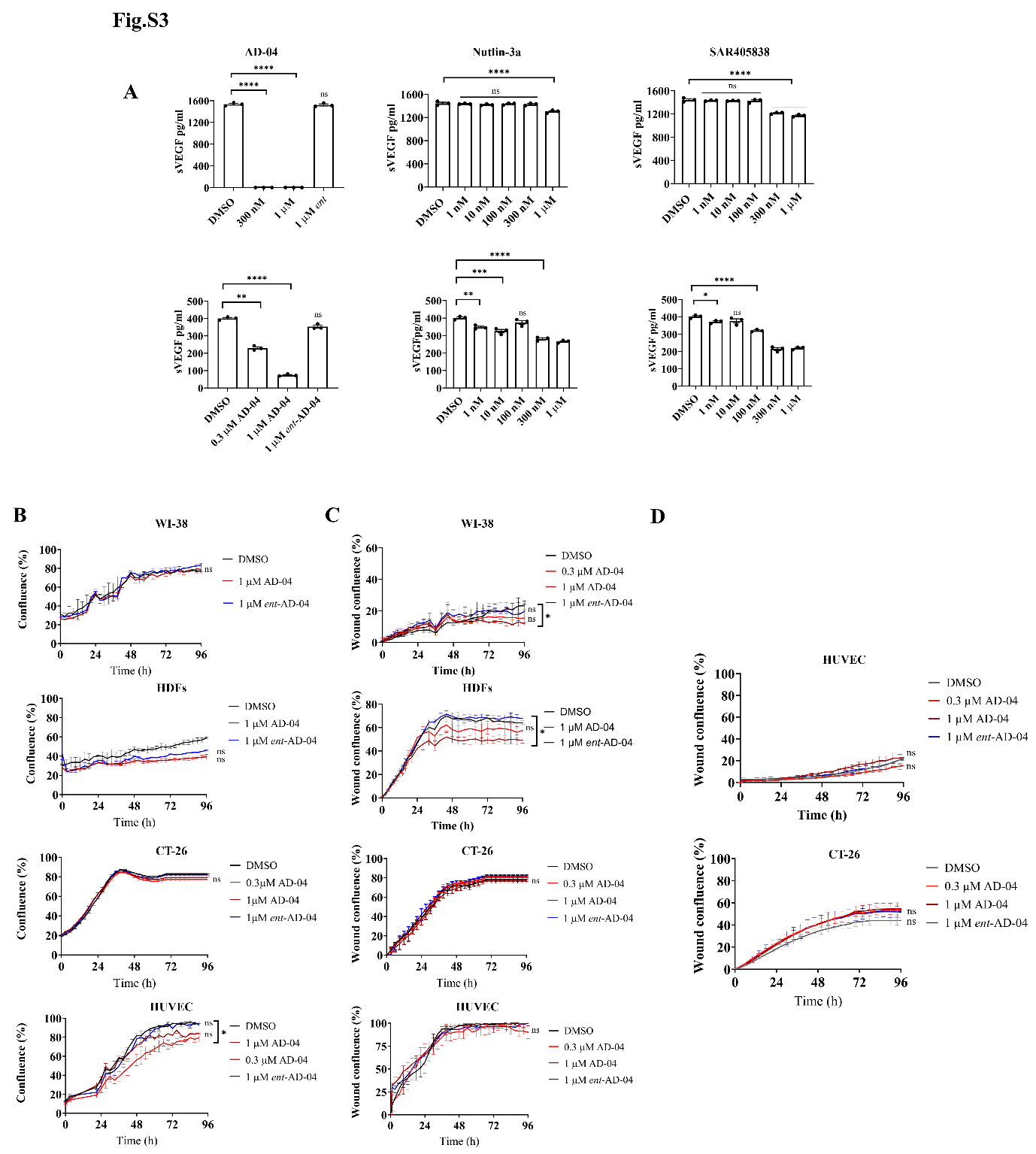
**

**Figure S3. Modulation of sVEGF, proliferation, migration and invasion in response to USP7 and MDM2 inhibitors.** (A) Cells were treated with AD-04, *ent*-AD-04, Nutlin-3a, or SAR405838 at indicated concentrations for 48h. Cell culture supernatants were collected and sVEGF measured by ELISA. Proliferation (B) or migration (C) of WI-38, HDFs, CT-26 and HUVEC cells treated with AD-04 or *ent*-AD-04. (D) HUVEC and CT-26 cells treated with AD-04 or *ent*-AD-04. Data presented as mean +/- S.E.M., **** p=<0.0001, *** p<0.001, ** p<0.01, *p=0.01, ns not significant.

**Supporting Information Figure S4.**

**
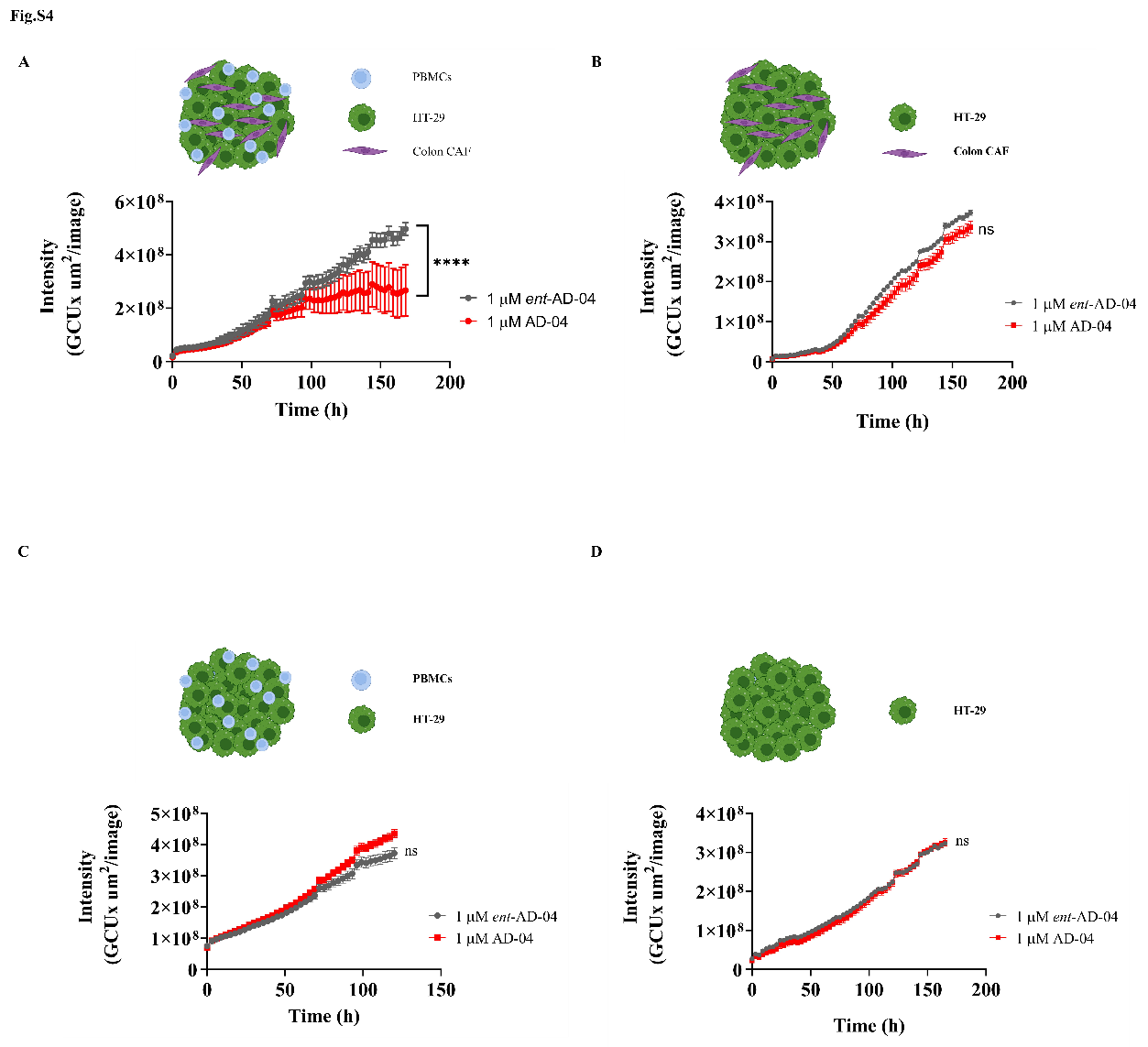
**

**
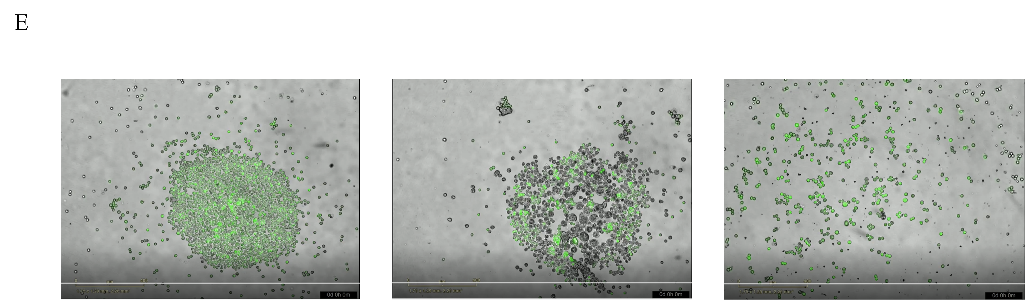
**

**Figure S4. Impact of USP7 inhibition on co-culture spheroid growth.** Spheroids formed from (A) HT-29-GFP cells, colon CAFs, and activated PBMCs, or (B) HT-29-GFP cells and colon CAFs, or (C) HT-29-GFP cells and activated PBMCs, or (D) HT-29-GFP cells alone, were treated with AD-04 or *ent*-AD-04. Data presented as mean +/- S.E.M., **** p=<0.0001 (ANOVA), ns not significant. (E) Additional MP4 videos illustrating: Spheroid formation of HT-29-GFP alone (video 1, left), HT-29-GFP + fibroblasts (video 2, middle), and HT-29-GFP + fibroblasts + PMBCs (video 3, right).

**Supporting Information Figure S5.**


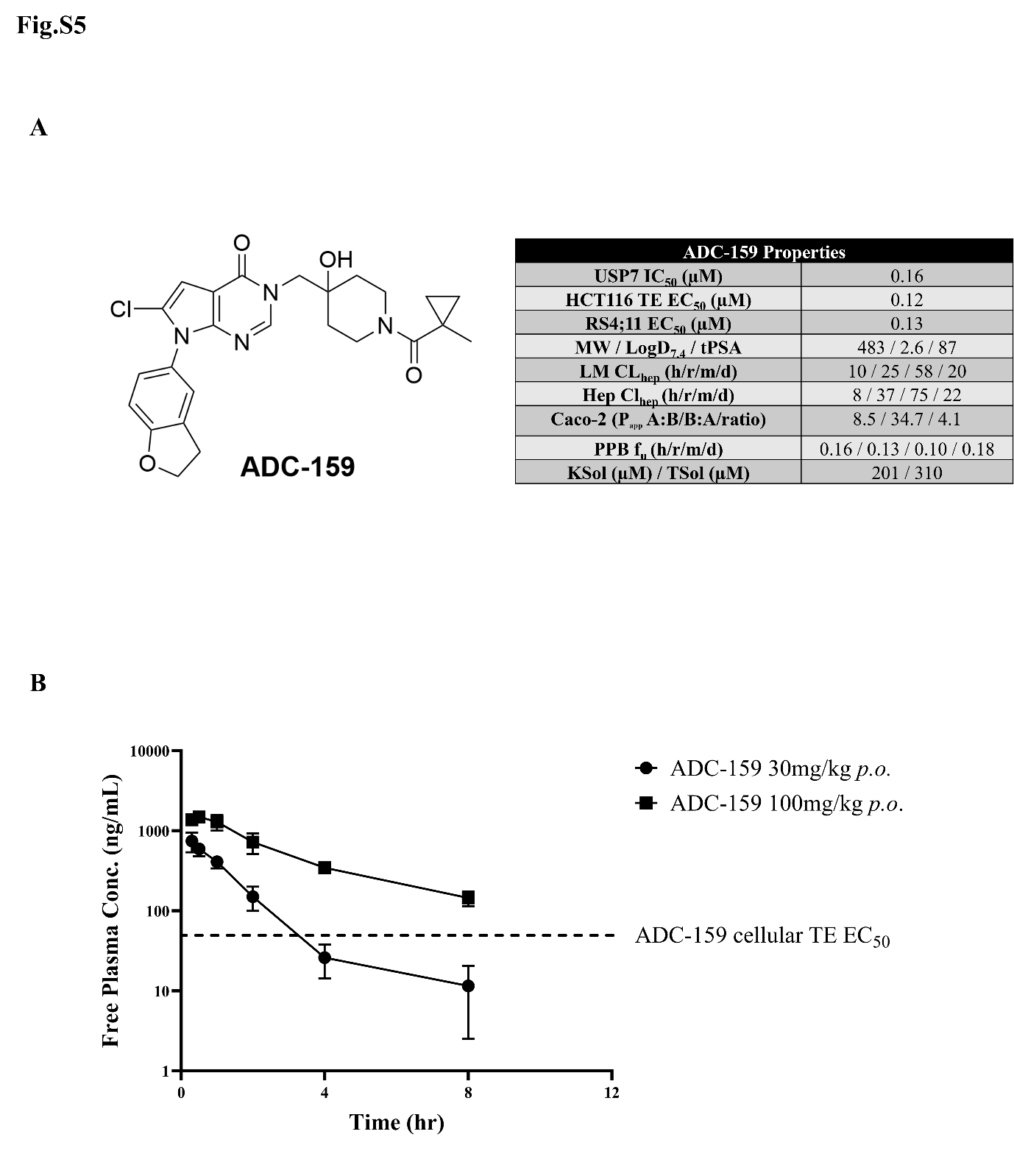


**Figure S5. Chemical structure, ADME properties and *in vitro* pharmacokinetic profile of ADC-159.** (A) Chemical structure and *in vitro* properties of orally bioavailable USP7 inhibitor, ADC-159. Target engagement (TE), molecular mass (MW), octanol/water coefficient at pH 7.4 (logD7.4), total polar surface area (tPSA), liver microsomal (LM CLhep) or hepatocyte (Hep CLhep) stability in human/rat/mouse/dog (h/r/m/d) (units mL/min/kg), permeability across Caco-2 cell monolayer from apical to basolateral (A to B) or basolateral to apical (B to A) directions (units 10^-6^ cm/s) and B:A/A:B efflux ratio, plasma protein binding fraction unbound (PPB fu), kinetic (KSol) or thermodynamic (TSol) solubility. (B) Concentration vs time curves following 30 mg/kg or 100 mg/kg single dose oral administration in BALB/c female mice. PK parameters were CLp=58 mL/min/kg, Vss = 1.1 (L/kg), t_1/2_ = 0.2h; the oral t is 1.5h and 2.9h for 30 mg/kg and 100 mg/kg, respectively. Doses of 30 mg/kg did not give sufficient free plasma concentrations and target engagement coverage for *in vivo* experiments.

**Supporting Information Figure S6.**


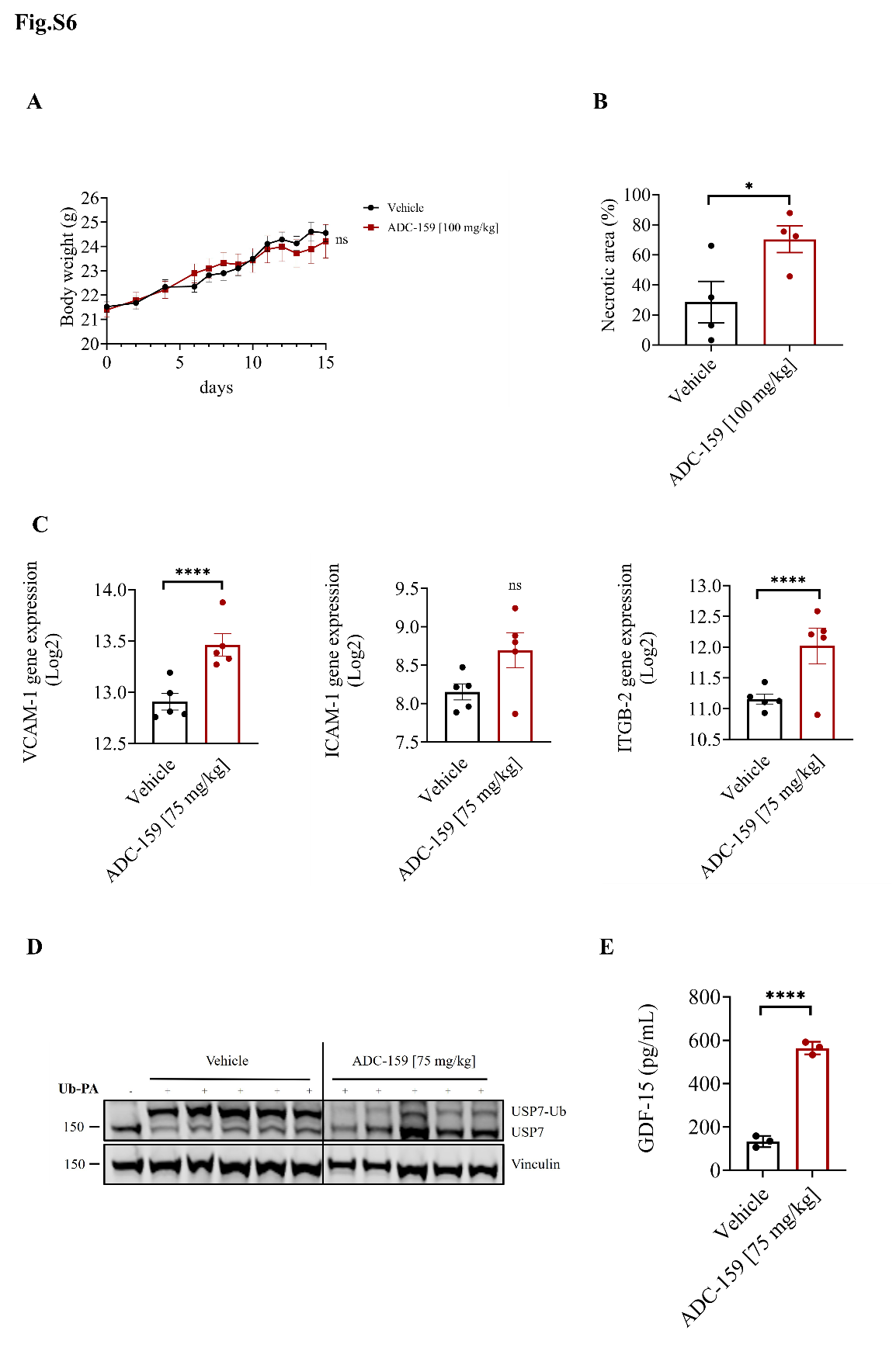


**Figure S6. Effect of USP7 on tumour microenvironment *in vivo*.** (A) Body weight changes of CT-26 tumor-bearing mice in each group (start of daily oral dosing shown with dotted line; day 0 = randomization and assignment to treatment groups). (B) Necrotic areas were quantified after 13 days of treatment with ADC-159 using HALO^®^ software. (C) Gene expression levels of endothelial-leukocyte interaction/adhesion/infiltration markers *ICAM-1*, *VCAM-1* and *ITGB-2* (n= 5). (D) Lysates from CT-26 tumors treated with ADC-159 for 13 days were incubated with ubiquitin-propargylamine (Ub-PA) activity probe. Samples were analyzed by immuno-blotting using USP7 antibody. (E) Serum samples from CT-26 tumor-bearing mice were collected after 13 days of treatment and circulating serum GDF-15 measured by ELISA (n=3 mice/group).


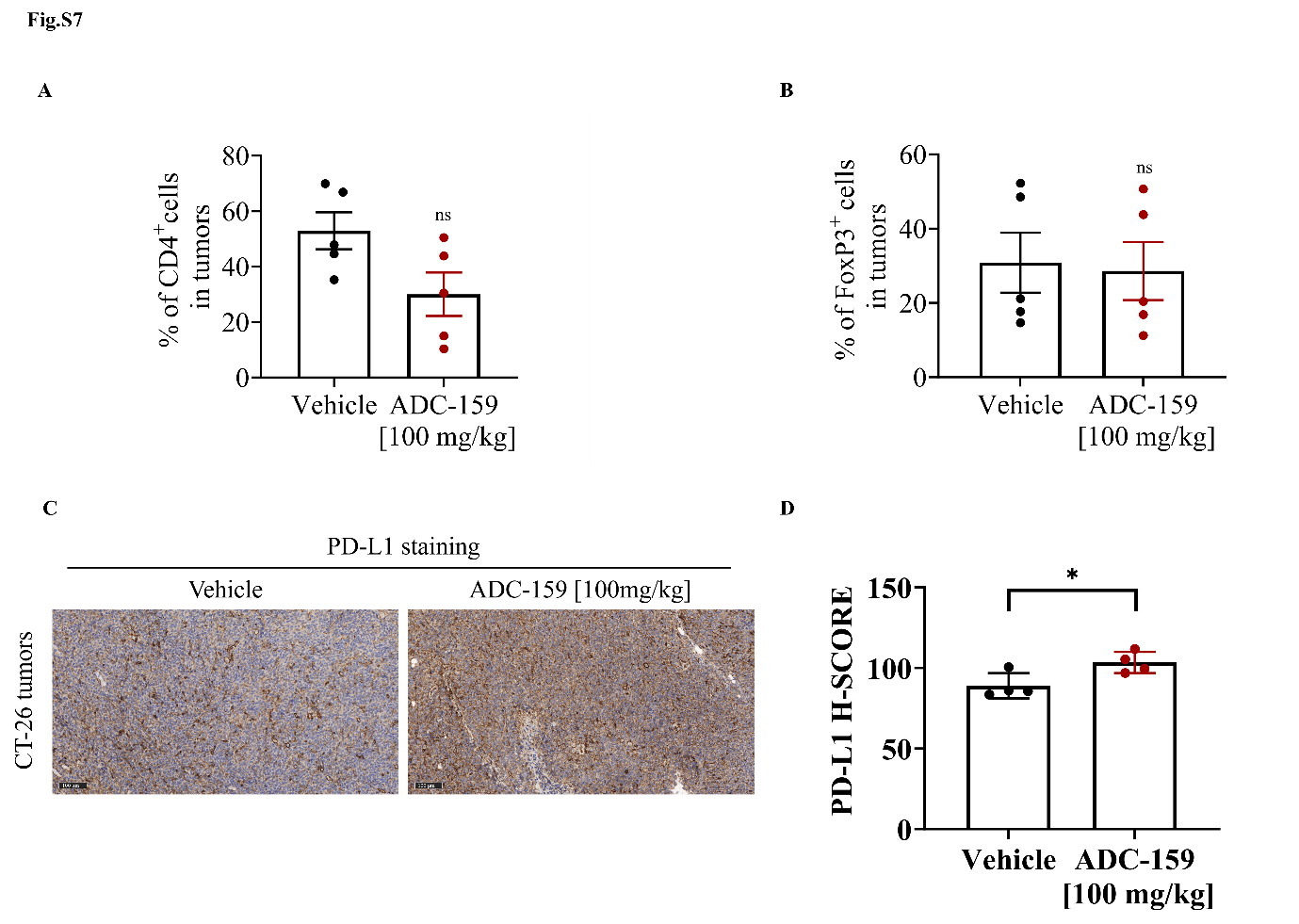
**Supporting Information Figure S7.**

**Figure S7. Impact of USP7 inhibitor on anti-tumor immunity.** (A) Intra-tumor CD4+ TILs monitored by FACS. (B) Percentage of Foxp3+ cells after 13 days of treatment with ADC-159 monitored by FACS. (C) PD-L1 expression in tumors treated with ADC-159 for 13 days was determined by IHC (x20 magnification, scale bar = 100 microns (Aperio ImageScope), and (D) scored using an H-score approach (described in Materials and Methods).
